# Supplementary material for: A scalable software solution for anonymizing high-dimensional biomedical data
Source: Gigascience. 2021 Oct 4;10(10):giab068. doi: 10.1093/gigascience/giab068 (PMC8489190; doi:10.1093/gigascience/giab068)

GigaScience

A scalable software solution for anonymizing high-dimensional biomedical data

--Manuscript Draft--

**Manuscript Number:**	GIGA-D-20-00292
**Full Title:**	A scalable software solution for anonymizing high-dimensional biomedical data
**Article Type:**	Technical Note
**Funding Information:**	
**Abstract:**	Background: Data anonymization is an important building block for ensuring privacy and fosters the re-use of data. However, transforming the data in a way it preserves the privacy of subjects while maintaining a high degree of data quality is challenging and particularly difficult when processing complex datasets that contain a high number of attributes. In this paper we present how we extended the open source software ARX to improve its support for high-dimensional, biomedical datasets.<br><br>Findings: For improving ARX's capability to find optimal transformations when processing high-dimensional data, we implement two novel search algorithms. The first one is a greedy top-down approach and is oriented on a formally implemented bottom-up search. The second is based on a genetic algorithm. We evaluated the algorithms with different datasets, transformation methods and privacy models. The novel algorithms mostly outperformed the previously implemented bottom-up search. Additionally, we extended the graphical user interface to provide a high degree auf usability and performance when working with high-dimensional datasets.<br><br>Conclusion: With our additions we have significantly enhanced ARX's ability to handle high-dimensional data in terms of processing performance as well as usability and thus can further facilitate data sharing.
**Corresponding Author:**	Thierry Meurers<br>Charite Universitatsmedizin Berlin<br>Berlin, GERMANY
**Corresponding Author Secondary Information:**	
**Corresponding Author's Institution:**	Charite Universitatsmedizin Berlin
**Corresponding Author's Secondary Institution:**	
**First Author:**	Thierry Meurers
**First Author Secondary Information:**	
**Order of Authors:**	Thierry Meurers
	Raffael Bild
	Kieu-Mi Do
	Fabian Prasser
**Order of Authors Secondary Information:**	
**Additional Information:**	
**Question**	**Response**
Are you submitting this manuscript to a special series or article collection?	No
**Experimental design and statistics**<br><br>Full details of the experimental design and	Yes

statistical methods used should be given in the Methods section, as detailed in our Minimum Standards Reporting Checklist. Information essential to interpreting the data presented should be made available in the figure legends.<br><br>Have you included all the information requested in your manuscript?	
**Resources**<br><br>A description of all resources used, including antibodies, cell lines, animals and software tools, with enough information to allow them to be uniquely identified, should be included in the Methods section. Authors are strongly encouraged to cite Research Resource Identifiers (RRIDs) for antibodies, model organisms and tools, where possible.<br><br>Have you included the information requested as detailed in our Minimum Standards Reporting Checklist?	Yes
**Availability of data and materials**<br><br>All datasets and code on which the conclusions of the paper rely must be either included in your submission or deposited in publicly available repositories (where available and ethically appropriate), referencing such data using a unique identifier in the references and in the "Availability of Data and Materials" section of your manuscript.<br><br>Have you have met the above requirement as detailed in our Minimum Standards Reporting Checklist?	Yes

Manuscript

Click here to access/download;Manuscript;High_Dim_Anonymization_f1_wo

A scalable software solution for anonymizing high-dimensional biomedical data

Thierry Meurers[1,2] (thierry.meurers@charite.de) (corresponding Author),

Raffael Bild[3] (raffael.bild@tum.de),

Kieu-Mi Do[4] (kieumi.do@tum.de),

Fabian Prasser[1,2] (fabian.prasser@charite.de)

1 Charité – Universitätsmedizin Berlin, corporate member of Freie Universität Berlin, Humboldt-Universität zu Berlin, and Berlin Institute of Health, Charitéplatz 1, 10117 Berlin, Germany

2 Berlin Institute of Health (BIH), Anna-Louisa-Karsch-Str. 2, 10178 Berlin, Germany

3 School of Medicine, Technical University of Munich, Ismaninger Str. 22, 81675 Munich, Germany

4 Faculty of Informatics, Technical University of Munich, Boltzmannstr. 3 85748 Garching, Germany

Abstract

Background: Data anonymization is an important building block for ensuring privacy and fosters the re-use of data. However, transforming the data in a way it preserves the privacy of subjects while maintaining a high degree of data quality is challenging and particularly difficult when processing complex datasets that contain a high number of attributes. In this paper we present how we extended the open source software ARX to improve its support for high-dimensional, biomedical datasets.

Findings: For improving ARX's capability to find optimal transformations when processing high-dimensional data, we implement two novel search algorithms. The first one is a greedy top-down approach and is oriented on a formally implemented bottom-up search. The second is based on a genetic algorithm. We evaluated the algorithms with different datasets, transformation methods and privacy models. The novel algorithms mostly outperformed the previously implemented bottom-up search. Additionally, we extended the graphical user interface to provide a high degree auf usability and performance when working with high-dimensional datasets.

Conclusion: With our additions we have significantly enhanced ARX's ability to handle high-dimensional data in terms of processing performance as well as usability and thus can further facilitate data sharing.

Keywords

data privacy, anonymization, de-identification, heuristics, genetic algorithm, software tool

1 Introduction

Big data technologies and latest data science methods promise to be valuable tools for providing new insights into the development and course of diseases. These insights can be used to derive new preventive, diagnostic and therapeutic measures [1]. Implementing these methods in practice requires access to comprehensive, multi-level datasets of high quality. At a large scale, this can only be achieved

by fostering the reuse of data from different contexts and the sharing of data across institutional boundaries. The reuse of data is also in line with the FAIR (Findable, Accessible, Interoperable, Reusable) data principles and supports the reproducibility of research. However, in the context of biomedical research, sharing data is challenging as it is important to account for ethical aspects [2], privacy concerns as well as data protection laws like for example the US Health Insurance Portability and Accountability Act (HIPAA) [3] or the European General Data Protection Regulation (GDPR) [4].

One important building block for ensuring privacy is to provide safe data that minimizes disclosure risks [5]. This can be achieved by employing data anonymization techniques, that transform the data to mitigate privacy risks [6], [7]. Typically, the anonymization process is not limited to the removal of directly identifying attributes such as the name, telephone number or insurance id number. Instead, it must also account for attributes like the postal code, age and gender that could be combined to re-identify individuals or derive sensitive personal information [8], [9]. However, transforming the data will also have an impact on its usefulness and striking the right balance between privacy and data quality is challenging and particularly difficult when working with high-dimensional datasets that contain a high number of attributes. The complexity of this task is also demonstrated by several re-identification attacks [10], [11]. To mitigate risks and put anonymization in practice, tools that implement formal approaches based on mathematical and statistical models can be utilized. An example of such a tool is the open source software ARX [6], [12]. It is focused on biomedical data and has been mentioned in several official policies and guidelines [13]–[15], used in research projects [16]–[18], and enabled several data publishing activities [19]–[21].

Versions of ARX up to 3.8.0 were only able to process datasets with a limited number of attributes that could be used for de-anonymization (up to about 15). The reason for this were twofold: (1) the software only had limited support for anonymization algorithms able to process high-dimensional data, (2) the graphical user interface was not designed to work with datasets containing a high number of attributes. In this work, we describe our efforts to overcome these limitations by implementing

additional anonymization algorithms and extending ARX's user interface with additional views that simplify the management of high-dimensional data.

2 Materials and Methods

In this section, we will first provide some fundamental details about data anonymization. Second, we will present important properties of the ARX Anonymization Tool that had an influence on our design decisions. Third, we will present the extensions implemented into ARX. Finally, we will provide insights into our experimental setup.

2.1 Fundamentals of Data Anonymization

When anonymizing a dataset the first step is to remove all attributes that directly identify the individuals. Thereafter, the dataset is modified or noise is introduced so that the risk of identified or identifiable individuals of being linked to one or multiple records of the dataset or to sensitive information in general is lowered [7]. This step involves the usage of mathematical or statistical privacy models used to quantify the risk of privacy breaches as well as quality models that measure the usefulness of the output data. For (1) measuring privacy risks, (2) measuring data quality and (3) transforming the data a variety of models can be employed and combined.

< Figure 1 >

Figure 1: Exemplary anonymization process.

Figure 1 shows a simplified example of an anonymization process. The transformation involves different procedures such as (1) randomly sampling the records, (2) aggregating values by replacing them with their mean, (3) suppressing values, (4) masking trailing characters of strings, (5) categorizing numerical values and (6) generalizing categorical attributes. These transformations may reduce the fidelity of the data but also reduce the risk of linkage attacks and the attacker's accuracy when linking records. Furthermore, an additional uncertainty could be created by introducing noise. The

transformed output data of the example fulfills two frequently used privacy models: k-Anonymity with k = 3 [22] and (ε, δ)-Differential Privacy with ε ≈ 0.92 and δ ≈ 0.22 [23].

The simple example demonstrates the variety of possibilities available for transforming the data. Furthermore, it also suggests why it is often not feasible to search the entire solution space of all potential output datasets when processing more complex data. For this kind of tasks, solutions that try to determine a good transformation scheme on a best-effort basis e.g. based on heuristic strategies [24]–[26] or clustering algorithms [27]–[29] have been developed. An overview of the solutions is provided by Fung et al. [7].

2.2 The ARX Anonymization Tool

ARX supports a variety of privacy models, quality models and data transformation schemes and allows for their arbitrary combination [6]. For transforming the data, it relies on domain generalization hierarchies which describe how values can be transformed to make them less unique. For each hierarchy it is possible to define multiple levels of generalization that cover an increasing range of the attribute's domain.

< Figure 2 >

Figure 2: Generalization hierarchies (a) and structure of the solution space (b) used by ARX.

The basic solution space that is utilized by ARX is given by all possible combinations of generalization levels defined by the hierarchies. An example is provided in Figure 2. Each possible generalization is called a generalization scheme. Mathematically, the solution space is a lattice [30], [31], which grows exponentially in size regarding the number of attributes that need to be protected [25]. As ARX is also able to apply different generalization schemes automatically to different parts of the input dataset the size of the solution space may grow further by a multiplicative factor representing the number of rows [6]. ARX supports different algorithms for finding optimal solutions within solution spaces of tractable size [32] as well as a heuristic algorithm for larger search spaces that tries to determine a good transformation scheme on a best-effort basis [25].

In addition to its anonymization engine, ARX also features a cross-platform graphical user interface. An overview of the different perspectives provided by the platform is shown in Figure 3.

< Figure 3 >

Figure 3: Basic perspectives of the graphical interface of the ARX Data Anonymization Tool.

In the *configuration* perspective it is possible to define risk thresholds for different types of attacks, to prioritize attributes by importance, to model the background knowledge of possible attackers and to define transformation methods and rules. In the *exploration* perspective, relevant anonymization strategies are visualized for the input data and a categorization according to output data quality is supported. A further perspective supports the manual *quality analysis* of the output data. Different methods for measuring the information content of the output data, descriptive statistics and methods for comparing the usefulness of the input and output data for different application scenarios are provided. In a *risk analysis* perspective, it is possible to visually compare input and output data using different risk models. However, in the user interface it is challenging to support high-dimensional datasets. For example, several perspectives and views of the software display lists of all attributes of the dataset loaded, which can become confusing and lead to performance problems on some platforms with an increasing number of attributes.

2.3 Integrating Anonymization Algorithms for High-Dimensional Data

As mentioned before, the anonymization procedures supported by ARX are built around a basic operator that searches through the generalization lattice. In prior work we have already integrated a greedy best-first bottom-up search algorithm into the software [25]. This algorithm starts at the bottom generalization scheme, which applies no generalization to the data. It then “expands” this generalization scheme, by applying all generalization schemes to the input dataset that can be derived by increasing one of the generalization levels. The quality of the resulting output dataset is computed for all these schemes, and the process is repeated by expanding the generalization resulting in the dataset with highest quality. This process is then repeated until a user-specified period of time has

passed. During the execution of the algorithm, a list of all generalization schemes that have been evaluated is stored and in each iteration, the scheme with the highest output data quality that has not yet been expanded is expanded. For further details we refer interesting readers to the original publication [25].

It must be noted that this process is only suitable for processing dataset of medium dimensionality (about 15 attributes) for several reasons. First, the search process may become trapped in local minima, as there is no significant diversification of the solutions considered. Second, the process naturally favors transformation schemes located in the lower part of the search space (i.e. schemes that apply a low degree of generalization). While this makes sense for anonymization processes that only apply generalization, the method reaches its limits with the complex transformation operations supported in newer versions of ARX in which different transformation schemas are used to transform different parts of a dataset. In this case, a better overall solution can sometimes be determined if outliers are transformed more strongly.

For this reason, we have integrated two new algorithms for processing high-dimensional data into the software.

The first algorithm closely resembles the bottom-up greedy best-first search but performs this process top-down. We will not describe it in further detail, as this is a straight-forward extension of the process described in the previous paragraphs.

The second algorithm aims to support diversification of the solutions considered, by applying a genetic optimization process to the anonymization problem. Genetic algorithms search for solutions in a heuristic manner that is oriented on the process of natural selection [33]. During the search, the solutions are considered chromosomes or individuals that carry the solution's properties encoded as a list of genes. The set of candidate solutions/individuals is called population. Mostly, the initial population is created by randomly generating individuals. Thereafter, the algorithm works iteratively. By crossing and mutating the individuals contained in the population each iteration will result in a new,

so-called, generation. Whether and how an individual is altered is determined by its fitness which usually is calculated using the cost function of the investigated optimization problem. Once reaching a predefined limit of iterations the fittest individual is considered the optimal solution. However, there is no guarantee that a globally optimal solution can always be found.

The genetic algorithm implemented into ARX is based on the work of Wan et al. [34]. Wan employed the algorithm for anonymization genomic data using a game-theoretic approach. As we have already successfully adapted and integrated the game-theoretic privacy model into ARX in prior work [35], we decided to also integrate the genetic search process into the software.

For our work we significantly modified the algorithm to make it compatible with the types of solution spaces used by ARX and to integrate it with the privacy and quality models supported by the software. Instead of a binary string every individual carries a list of numerical values representing a generalization scheme. The list's length equals the number of attributes that need to be transformed and the i-th value of the list represents the generalization level of the i-th attribute. When generating new individuals or altering single genes we choose a random value in between the lowest and highest generalization level available for the corresponding attribute. The populations are implemented in a matrix like structure with the rows of this matrix representing individuals (generalization scheme) and columns their genes (generalization level of an attribute). ARX's privacy and quality models have been integrated via the fitness function. ARX always automatically alters the output of any given transformation in such a way that the required privacy guarantees are provided. This is achieved by suppressing records [36]. The suppression of records is captured by a decrease in data quality. Hence, we defined the fitness of a transformation to equal output data quality, which not only measures the transformation's direct impact on data quality but also implicitly captures how well the required privacy guarantees are achieved.

The algorithm itself works as follows:

- **Initialization:** During the initialization two equally sized subpopulations are created. The first individuals of the first subpopulation are generated following a "triangle" pattern using the lowest and highest generalization levels to cover the solution space. An example is provided in Figure 4. The remaining individuals of the first subpopulation as well as the entire second subpopulation is filled by randomly creating individuals.
- **Iteration:** After initializing the subpopulations the algorithm's main loop is started. The algorithm stops after reaching a pre-defined number of iterations or time limit. Within the loop the following steps are executed:

  Step 1: Sorting: The individuals contained in the subpopulations are sorted by their fitness in descending order.

  Step 2: Selection: The fittest individuals of the current population will simply be copied to the next generation without being modified. We refer to this fraction of individuals as *elite fraction*.

  Step 3: Crossover: Next, the so-called *crossover fraction* of the new generation is populated. For this purpose, two parent-individuals from the *production fraction* of the current population are crossed to generate a new child-individual. The probability of being chosen as a parent increases with the fitness. The crossover is performed in a randomized fashion. For every gene it is decided randomly from which of the two parents it is inherited.

  Step 4: Mutation: The rest of the new generation is populated by randomly choosing individuals of the current generation and mutating them by altering their genes. The number of changed genes is randomly chosen between 1 and an upper bound which is calculated by multiplying the *mutation probability* with the number of available genes.

  Step 5: Swapping: Additionally, it is possible that the fittest individuals are swapped between the two subpopulations. How often they are changed depends on the *immigration interval* which refers to the number of iterations between the swaps. The number of exchanged individuals can be controlled by the *immigration fraction*.

< Figure 4 >

Figure 4: Initialization of the first subpopulation for a solution space with the highest generalization levels of [3,1,5,3,1].

2.4 Extending the User-Interface for High-Dimensional Data

ARX is implemented as a cross-platform program using Java and executed on the Java Virtual Machine. The Graphical User Interface (GUI) is implemented using the Standard Widget Toolkit (SWT), which enables implementing native GUIs on three supported platforms: Windows, Linux and MacOS.

For improving the GUI's usability when working with high-dimensional datasets we made use of two SWT-based components provided by the Eclipse Nebula Project [37]. The first is NatTable. Based on the idea of virtual tables it ensures that the GUI remains responsive and provides a high rendering performance when displaying large datasets. The second is Pagination Control. This component is used to display a navigation page when working with tables used to configure a potentially large number of attributes.

Additionally, ARX features a mechanism that automatically detects the type of an attribute to ease the initial import of data as well as the ability to configure multiple attributes at once. These last two features are also available for smaller dataset but are especially helpful when working with high-dimensional datasets.

2.5 Experimental Design

2.5.1 Experiments

With the extensions described in this article, ARX now supports three algorithms for anonymizing high-dimensional data: (1) our initial bottom-up search, (2) the new top-down search and (3) the new genetic search algorithm. We performed a series of experiments, to study how well these algorithms work for different types of data to provide users with insights into which algorithm should be used in which context. In total, we conducted two experiments:

(1) **Low dimensional data:** We compared the algorithms to the optimal algorithm already supported by ARX [32] in the low-dimensional setting. We did this for two reasons. First, heuristic algorithms might also be relevant when anonymizing low-dimensional data if they significantly outperform optimal algorithms in terms of the time needed to find the optimal solution. Second, experiments with low-dimensional data might provide insights into basic strengths and weaknesses of the approaches. To this end, we compared the overall execution time of ARX's optimal algorithm with the time needed by the heuristic algorithms to find the optimal solution.

(2) **High-dimensional data:** Here, we use the three heuristic algorithms to anonymize high-dimensional datasets. This experiment was performed to determine whether the novel approaches (genetic and top-down) offer an advantage over the bottom-up algorithm. To this end, we executed the algorithms with different time limits and compared the quality of their results.

2.5.2 Privacy, quality and transformation model

To investigate a broad spectrum of anonymization problems, we decided to utilize different privacy and data transformation models.

For measuring and managing privacy risks, we used two models:

(1) **Distinguishability:** To implement restrictions on the distinguishability of data, we utilized the well-known and relatively strict k-anonymity model. A dataset is k-anonymous if every record cannot be distinguished from at least k-1 other records in respect to attributes that may be used to de-anonymize the data [38]. As a parameter we used k=5 which is a common recommendation [39].

(2) **Population uniqueness:** ARX also supports statistical models that estimate disclosure risks by estimating the fraction of records in a dataset that are expected to be unique in the overall population. Compared to k-anonymity, this is a relatively weak privacy model. For our

experiments we enforced a uniqueness of 1 % within the US population and relied on the model introduced by Pitman to estimate population characteristics [40], [41].

For transforming data, we also used two common models:

(1) **Global generalization**: With this model, the values in a dataset are generalized based on user-defined hierarchies. In this process, it is guaranteed that all values of an attribute are generalization to the same level of the associated hierarchy. To prevent overgeneralization, records can also be removed from the dataset.

(2) **Local generalization:** With this model, data is also transformed by generalization, but values of the same attribute in different records can be transformed differently. Records may also be removed, but this is typically not required due to the flexibility of the transformation model.

In ARX, local transformations are implemented by using an iterative process in which the dataset is automatically partitioned and different transformation schemes are applied to different partitions [6]. In our experiments with local generalization we used 100 iterations and different time limits for individual iterations.

To quantify data quality, we decided to use the intuitive "Granularity" model [42], which measures the value-level precision of the output data. The measurements are normalized with 0 % representing a dataset from which all information has been removed and 100 % corresponding to a completely unmodified dataset [6].

2.5.3 Parameterization

While the top-down and bottom-up search algorithms do not require any additional parameterization, the genetic search algorithm features multiple configuration parameters, which are shown in Table 1.

Table 1: Parameters of the genetic algorithm and the values employed in the experiments.

Parameter	Description	Value
Elite fraction	Fraction of individuals that is directly copied to the next generation.	0.2
Crossover fraction	Fraction of individuals that is replaced by new individuals that are generated by crossing two parents from the production fraction.	0.4
Production fraction	Fraction of individuals used as parents when generating crossover individuals.	0.2
Mutation probability	Used to calculate the upper bound of changed genes when mutating individuals.	0.05
Immigration fraction	The fraction of individuals that is swapped between the subpopulation.	0.2
Immigration Interval	Number of iterations between swaps.	10
Iterations	Number of iterations performed by the GA	50
Subpopulation size	Number of individuals contained in each of the subpopulations.	100

In ARX, these parameters are presented as configuration options to the users. For our experiments we parameterized the algorithm following the suggestions by Wan et al. [34] with the only exception being the production fraction which we set to 0.2. By only selecting the top 20 % of individuals as parents we can fasten the process of finding an optimal solution. In contrast, Wan et al. set the production fraction to 0.8 which makes the algorithm less prone to local minima.

2.5.4 Technical Setup

We repeated each experiment five times and report the average for two reasons: first, it is well known that execution times of JVM-based programs vary slightly due to effects from functionalities, such as just-in-time compilation. Second, the genetic algorithm is randomized and hence may perform slightly different in each execution.

The experiments were performed on a desktop computer with an AMD Ryzen 2700X processor (8 cores, 3.7-4.3 GHz) running 64-bit Windows 10 (version 1909) and a 64-bit Oracle JVM (version 1.8.0).

2.5.5 Datasets

For evaluating the performance of the heuristic algorithms, we used six different real-world datasets. An overview of the properties of the datasets is shown in Table 2. Most of them have already been utilized in previous evaluations of data anonymization algorithms.

As low-dimensional datasets we choose (1) an excerpt of the 1994 US census dataset (*Census income*) which can be considered the de-facto standard for evaluating anonymization algorithms, (2) data from a nationally representative U.S. time diary survey and (3) results from the integrated health interview series collecting data on the health of the U.S. population.

As high-dimensional datasets we included (1) data from the responses to the American Community Survey (ACS) which captures demographic, social and economic characteristics of people living in the U.S., (2) a credit card client dataset from Taiwan used to estimate costumers default payments and (3) answers to a psychological test designed to measure someone's Machiavellianism from the open-source psychometrics project. As attributes that needed to be transformed, we selected variables that are typically associated with a high risk of re-identification. These included demographic data, timestamps, spatial information, medical attributes and payment histories.

Table 2: Overview of the datasets used for comparing the algorithms.

Name	#Attributes	#Records	Solution space size	Category
Census income [43]	9	30,162	12,960	Low dimensional
Time use [44]	9	539,254	34,992	Low dimensional
Health interviews [45]	9	1,185,424	25,920	Low dimensional
Census community [46]	30	68,725	203,843,174,400	High dimensional

Credit card [47]	24	30,000	49,478,023,249,920	High dimensional
Psychology test [48]	16	73,489	85,030,560	High dimensional

3 Results

3.1 Experimental results

3.1.1 Low-dimensional data

The results of the first set of experiments are displayed in Figure 5. For each heuristic algorithm, it shows the time in seconds needed to determine the optimal solution (and the overall execution time for the optimal algorithm) using the global transformation model. We did not use the local transformation model in this experiment, as the underlying algorithm is heuristic in nature (independently of the actual search strategy used) and can therefore not be used to compare the time needed to achieve a specific result in terms of output data quality [6].

< Figure 5 >

Figure 5: Time required for finding an optimal solution for different low-dimensional datasets using global generalization.

As can be seen, heuristic approaches provided a valuable alternative to the optimal approach even in low-dimensional settings. When aiming for a threshold on distinguishability, the bottom-up and top-down search algorithms almost always outperformed the optimal algorithm. On average, the genetic algorithm was slower than the other heuristic approaches, because it aims at diversifying the solutions considered, which is not a desirable feature in low-dimensional settings. Whether the top-down approach or the bottom-up approach performed better was associated with the degree of generalization required and hence with the fact whether the optimal solution is located more closely to the top or to the bottom of the lattice.

When optimizing for a threshold on population uniqueness the optimal algorithm outperformed the heuristic approaches in two out of three cases. This can be explained by the fact that calculating population uniqueness is much more computationally complex than checking for k-anonymity, as bivariate non-linear equation systems need to be solved. As a consequence, execution times are not dominated by the time needed to transform the dataset but by the time needed to evaluate the privacy model. The optimal approach implements a wide variety of pruning strategies that reduce the number of transformations that need to be checked [36], which cannot be implemented by the heuristic algorithms. The genetic algorithm provided the worst overall performance, as it tries to look at a diverse set of potential solutions.

3.1.2 High-dimensional data

The results of the experiments with high-dimensional data are displayed in Figure 6 and Figure 7. We compared the development of output data quality for the different algorithms over time and present two different types of results. For global transformation we continuously measured the development of output data quality over time. For local transformation we present the output data quality achieved with different time limits as the heuristic nature of the local transformation algorithm implemented in ARX makes it difficult to directly track the progress [6].

< Figure 6 >

Figure 6: Quality improvement over time for different high-dimensional datasets using global generalization.

Figure 6 shows the development of output data quality over time when using the global transformation model until the results of all three algorithms stabilized. As can be seen, all algorithms almost always eventually found a solution with comparable quality. However, when enforcing a threshold on population uniqueness on the credit card dataset, the bottom-up algorithm exhibited sub-optimal performance. Moreover, in most cases the genetic and top-down approach found better solutions much quicker than the bottom-up algorithm. When comparing the different algorithms to each other it can be seen that the genetic algorithm was generally good at quickly determining a relatively good

solution while the top-down algorithm provided a good balance of optimization speed and quality of its overall output. It can also be seen that output data quality was higher when reducing population uniqueness compared to reducing distinguishability, as the former model is weaker than the latter (see Section 2.5.2).

Figure 7 provides additional insights by presenting the results for the local transformation model.

< Figure 7 >

Figure 7: Achieved quality for different high-dimensional datasets using local generalization.

Again, the time axis covers the time that was needed for the solutions of the different algorithms to stabilize. As can be seen, the results are quite similar to the results obtained using the global transformation model, apart from the fact that the overall output data quality is higher with this transformation method. The genetic algorithm is good at very quickly finding a relatively good transformation and in most cases all algorithms finally found a comparable solution. The credit card dataset is a notable exception. In this case, the bottom-up algorithm provided the best result when reducing population uniqueness and the top-down approach provided the best result when reducing distinguishability. It is notable that the genetic algorithm performed best for short time limits in the former case, as the credit card dataset results in the largest solution space and the evaluation of individual solution candidates is expensive for population uniqueness. Moreover, good solutions were not located close to the top or bottom of the search space. This is exactly the scenario in which one would expect good performance from a genetic search process.

3.2 Extended User-Interface

In the updated version of the ARX GUI, seven views of the software distributed over all four perspectives have been extended using the pagination feature. We note that this extension is graceful, meaning that it is only activated when a high-dimensional dataset is loaded into the software (an according threshold can be specified in the tool's settings). As an example, the pagination feature of a view in ARX's quality analysis perspective is shown in Figure 8.

< Figure 8 >

Figure 8: Screenshots from the "Classification model" tab before (left) and after (right) adding the pagination feature.

Further features that are important for managing high-dimensional data with ARX , such as auto-detection of data types and options to configure multiple attributes at once, are located in different parts of the GUI, such as data import and hierarchy creation wizards as well as the software's main toolbar.

4 Discussion

4.1 Principal results

In this paper we presented the results of our efforts to improve the ability of the ARX Anonymization Tool to handle high-dimensional data. For this purpose, we extended the graphical user interface and introduced and evaluated two new heuristic anonymization algorithms.

The results of our evaluation showed that the two new algorithms outperform the heuristic algorithm previously used by ARX. However, no solution performed well in all cases. Therefore, it is important to provide multiple algorithms to users so that they can obtain high-quality output data in many scenarios. In this regard it is important that the newly implemented heuristic algorithms, top-down and genetic search, follow completely different concepts. The former approach complements the existing greedy algorithm while the latter approach aims at diversifying the potential solutions considered using the process of natural selection. Our results with low-dimensional data also show that the new algorithms can be helpful to improve computational efficiency even in scenarios were optimal algorithms could be used.

4.2 Comparison with Prior Work

Our work is not the first to focus on genetic algorithms for data anonymization. First, there is the algorithm by Wan et al. [34], which we have adopted in our work and described in detail in Section 2.3. Second, genetic algorithms have also been used in clustering-based anonymization processes. To

reduce distinguishability, such algorithms partition the records of a dataset into several groups with each of the groups containing at least k members, hence implementing the k-anonymity model. Similar records (regarding the values of attributes that may be used for de-anonymization) are placed in the same group. After the partitioning step, all records are modified in a manner that makes them indistinguishable from the other records in their group. Therefore, it is important to maximize homogeneity within the groups to reduce the overall loss of information. Solanas et al. [49] demonstrated how the computationally challenging partition step can be performed using a genetic algorithm. In their approach, the number of genes equals the number of records in the dataset with the i-th gene representing the group of the i-th record. The groups are encoded as an alphabet with a fixed size as the maximal number of different groups can be derived from k and the number of records in the dataset.

Lin et al. [27] described how the scalability of the clustering process can be improved for large datasets. Instead of the commonly used Pittsburgh approach (were each chromosome represents a complete solution) the Michigan approach was employed. Using this approach, the solution is encoded by the entire population and not by a single chromosome.

Iyengar [42] has demonstrated how a genetic algorithm can be used to determine intervals for generalizing values. In simplified terms the chromosome is a binary string with a length derived from the number of processed attributes and the number of their distinct values. A value of “1” in the chromosome implies that a value is used as an interval boundary.

More problem specific applications of genetic algorithms in the context anonymization include the anonymization of graphs [50].

Heuristics anonymization algorithms comparable to the bottom-up approach evaluated in our paper include DataFly [24] and iGreedy [26]. Both use global generalization and are focused on k-anonymity only. They are based on a bottom-up search and follow the concept of minimal anonymization meaning they terminate as soon as they find a transformation that fulfills the requested privacy properties. In previous work we have already shown that the bottom-up algorithm implemented by ARX outperforms

these approaches [25]. Furthermore, other researchers have focused on top-down search strategies. Important examples include the work of He et al. [51] who proposed a greedy top-down algorithm to partition a dataset and apply local generalization as well as the Top-Down Specialization method described by Fung et al. that iteratively specializes attributes until violating the anonymity requirements [52].

5 Conclusion and future work

With the work presented in this article we have significantly enhanced ARX's ability to handle high-dimensional data, both in the GUI and the Application Programming Interface (API). All features described in this article are available as open source software and will be included in the next release of the software [12].

In future work, we plan to add additional features to improve ARX's performance for high-dimensional data. While ARX already supports a wide range of data transformation models, we believe that the addition of further transformation methods would have the largest impact. One important example is sub-tree generalization, which provides a good balance between improved output data quality and interpretability of output datasets [53]. Moreover, we plan to add further methods from the area of statistical disclosure control, such as Post-Randomization (PRAM), that can be used to inject uncertainty into data with little impact on its usefulness [54].

Availability of source code and requirements

Project name: ARX Anonymization Tool

Project home page: https://arx.deidentifier.org/

GitHub repository: https://github.com/arx-deidentifier/arx

Operating system(s): Platform independent

Programming language: Java 8

Other requirements: None

License: Apache License 2.0

Project name: Benchmark of ARX's Heuristic Algorithms

GitHub repository: https://github.com/arx-deidentifier/genetic-benchmark

Operating system(s): Platform independent

Programming language: Java 8, Python 3

Other requirements: None

License: Apache License 2.0

Availability of supporting data

The datasets used to benchmark the algorithms are publicly available. The corresponding download URLs are referenced in Table 2 in Section 2.5.5. Additionally, the datasets are part of the GitHub repository of the Benchmark project (https://github.com/arx-deidentifier/genetic-benchmark). The repository also contains the generalization hierarchies used for anonymizing the data and the raw benchmark results as .csv files.

Competing interests

The authors declare that there is no conflict of interest.

Funding

The authors received no specific funding for this work.

Author Contributions

R.B. initiated and conceptualized the work. F.P., K.D. and T.M. implemented the novel anonymization algorithms and integrated them into ARX. F.P. and T.M. reworked the user-interface of ARX. T.M. programmed the framework used to evaluate the novel algorithms and performed the benchmarks.

T.M. and F.P. drafted the manuscript. R.B. and K.D. revised the manuscript and provided important suggestions for further improvements. All authors read and approved the final manuscript.

References

[1] S. Schneeweiss, “Learning from Big Health Care Data,” *N. Engl. J. Med.*, vol. 370, no. 23, pp. 2161–2163, Jun. 2014, doi: 10.1056/NEJMp1401111.

[2] A. Ballantyne, “Where is the human in the data? A guide to ethical data use,” *GigaScience*, vol. 7, no. 7, Jul. 2018, doi: 10.1093/gigascience/giy076.

[3] Office for Civil Rights, HHS, “Standards for privacy of individually identifiable health information. Final rule,” *Fed. Regist.*, vol. 67, no. 157, pp. 53181–53273, Aug. 2002.

[4] European Parliament and Council of the European Union, “Regulation (EU) 2016/679 of the European Parliament and of the Council of 27 April 2016 on the protection of natural persons with regard to the processing of personal data and on the free movement of such data, and repealing Directive 95/46/EC (General Data Protection Regulation),” *Regul. EU*, vol. 679, p. 2016, 2016.

[5] F. Ritchie, “Five Safes: designing data access for research,” 2016, doi: 10.13140/RG.2.1.3661.1604.

[6] F. Prasser, J. Eicher, H. Spengler, R. Bild, and K. A. Kuhn, “Flexible data anonymization using ARX—Current status and challenges ahead,” *Softw. Pract. Exp.*, vol. 50, no. 7, pp. 1277–1304, Jul. 2020, doi: 10.1002/spe.2812.

[7] B. Fung, K. Wang, A. W.-C. Fu, and P. Yu, *Introduction to privacy-preserving data publishing: Concepts and techniques*. 2010, p. 341.

[8] L. Rocher, J. M. Hendrickx, and Y.-A. de Montjoye, “Estimating the success of re-identifications in incomplete datasets using generative models,” *Nat. Commun.*, vol. 10, no. 1, p. 3069, Dec. 2019, doi: 10.1038/s41467-019-10933-3.

[9] L. Sweeney, “Simple Demographics Often Identify People Uniquely,” *Carnegie Mellon University, Data Privacy*, 2000.

[10] K. El Emam, E. Jonker, L. Arbuckle, and B. Malin, “A Systematic Review of Re-Identification Attacks on Health Data,” *PLoS ONE*, vol. 6, no. 12, p. e28071, Dec. 2011, doi: 10.1371/journal.pone.0028071.

[11] J. Henriksen-Bulmer and S. Jeary, “Re-identification attacks—A systematic literature review,” *Int. J. Inf. Manag.*, vol. 36, no. 6, pp. 1184–1192, Dec. 2016, doi: 10.1016/j.ijinfomgt.2016.08.002.

[12] F. Prasser, “ARX - Data Anonymization Tool | A comprehensive software for privacy-preserving microdata publishing,” 2020. https://arx.deidentifier.org/ (accessed Jul. 08, 2020).

[13] “OLD External guidance on the implementation of the European Medicines Agency policy on the publication of clinical data for medicinal products for human use.” Nov. 09, 2018.

[14] Personal Data Protection Commission of Singapore, “The anonymisation decision-making framework.” Jan. 2018.

[15] M. Elliot, E. Mackey, K. O’Hara, and C. Tudor, *The Anonymisation Decision-Making Framework*. 2016.

[16] Lei Xu, Chunxiao Jiang, Yan Chen, Yong Ren, and K. J. R. Liu, “Privacy or Utility in Data Collection? A Contract Theoretic Approach,” *IEEE J. Sel. Top. Signal Process.*, vol. 9, no. 7, pp. 1256–1269, Oct. 2015, doi: 10.1109/JSTSP.2015.2425798.

[17] J. Kim, H. Ha, B.-G. Chun, S. Yoon, and S. K. Cha, “Collaborative analytics for data silos,” in *2016 IEEE 32nd International Conference on Data Engineering (ICDE)*, Helsinki, Finland, May 2016, pp. 743–754, doi: 10.1109/ICDE.2016.7498286.

[18] C. Costa, G. Chatzimilioudis, D. Zeinalipour-Yazti, and M. F. Mokbel, “Efficient Exploration of Telco Big Data with Compression and Decaying,” in *2017 IEEE 33rd International Conference on Data Engineering (ICDE)*, San Diego, CA, USA, Apr. 2017, pp. 1332–1343, doi: 10.1109/ICDE.2017.175.

[19] J. Kuzilek, M. Hlosta, and Z. Zdrahal, “Open University Learning Analytics dataset,” *Sci. Data*, vol. 4, no. 1, Art. no. 1, Nov. 2017, doi: 10.1038/sdata.2017.171.

[20] G. Ursin, S. Sen, J.-M. Mottu, and M. Nygård, “Protecting Privacy in Large Datasets—First We Assess the Risk; Then We Fuzzy the Data,” *Cancer Epidemiol. Biomarkers Prev.*, vol. 26, Jul. 2017, doi: 10.1158/1055-9965.EPI-17-0172.

[21] “Lean European Open Survey on SARS-CoV-2 Infected Patients - Studying SARS-CoV-2 collectively,” *Lean European Open Survey on SARS-CoV-2 Infected Patients*. https://leoss.net/ (accessed Jul. 09, 2020).

[22] L. Sweeney, “ACHIEVING k-ANONYMITY PRIVACY PROTECTION USING GENERALIZATION AND SUPPRESSION,” *Int. J. Uncertain. Fuzziness Knowl.-Based Syst.*, vol. 10, no. 05, pp. 571–588, Oct. 2002, doi: 10.1142/S021848850200165X.

[23] C. Dwork and A. Roth, “The Algorithmic Foundations of Differential Privacy,” *Found. Trends® Theor. Comput. Sci.*, vol. 9, no. 3–4, pp. 211–407, 2013, doi: 10.1561/0400000042.

[24] L. Sweeney, “Datafly: a system for providing anonymity in medical data,” in *Database Security XI*, T. Y. Lin and S. Qian, Eds. Boston, MA: Springer US, 1998, pp. 356–381.

[25] F. Prasser, R. Bild, J. Eicher, H. Spengler, and K. A. Kuhn, “Lightning: Utility-Driven Anonymization of High-Dimensional Data,” p. 25, 2016.

[26] K. Babu, N. Ranabothu, N. Kumar, M. Elliot, and S. Jena, “Achieving k-anonymity Using Improved Greedy Heuristics for Very Large Relational Databases,” *Trans. Data Priv.*, vol. 6, pp. 1–17, Apr. 2013.

[27] J.-L. Lin and M.-C. Wei, “Genetic algorithm-based clustering approach for k-anonymization,” *Expert Syst. Appl.*, vol. 36, no. 6, pp. 9784–9792, Aug. 2009, doi: 10.1016/j.eswa.2009.02.009.

[28] J.-W. Byun, A. Kamra, E. Bertino, and N. Li, “Efficient k-Anonymization Using Clustering Techniques,” in *Advances in Databases: Concepts, Systems and Applications*, vol. 4443, R. Kotagiri, P. R. Krishna, M. Mohania, and E. Nantajeewarawat, Eds. Berlin, Heidelberg: Springer Berlin Heidelberg, 2007, pp. 188–200.

[29] G. Loukides and J. Shao, “Clustering-Based K-Anonymisation Algorithms,” in *Database and Expert Systems Applications*, vol. 4653, R. Wagner, N. Revell, and G. Pernul, Eds. Berlin, Heidelberg: Springer Berlin Heidelberg, 2007, pp. 761–771.

[30] K. El Emam *et al.*, “A Globally Optimal k-Anonymity Method for the De-Identification of Health Data,” *J. Am. Med. Inform. Assoc.*, vol. 16, no. 5, pp. 670–682, Sep. 2009, doi: 10.1197/jamia.M3144.

[31] F. Kohlmayer, F. Prasser, C. Eckert, A. Kemper, and K. A. Kuhn, “Highly efficient optimal k-anonymity for biomedical datasets,” in *2012 25th IEEE International Symposium on Computer-Based Medical Systems (CBMS)*, Rome, Italy, Jun. 2012, pp. 1–6, doi: 10.1109/CBMS.2012.6266366.

[32] F. Kohlmayer, F. Prasser, C. Eckert, A. Kemper, and K. A. Kuhn, “Flash: Efficient, Stable and Optimal K-Anonymity,” in *2012 International Conference on Privacy, Security, Risk and Trust and 2012 International Confernece on Social Computing*, Amsterdam, Netherlands, Sep. 2012, pp. 708–717, doi: 10.1109/SocialCom-PASSAT.2012.52.

[33] M. Mitchell, *An introduction to genetic algorithms*. Cambridge, Mass: MIT Press, 1996.

[34] Z. Wan, Y. Vorobeychik, W. Xia, E. W. Clayton, M. Kantarcioglu, and B. Malin, “Expanding Access to Large-Scale Genomic Data While Promoting Privacy: A Game Theoretic Approach,” *Am. J. Hum. Genet.*, vol. 100, no. 2, pp. 316–322, Feb. 2017, doi: 10.1016/j.ajhg.2016.12.002.

[35] F. Prasser *et al.*, “An Open Source Tool for Game Theoretic Health Data De-Identification,” *AMIA Annu. Symp. Proc. AMIA Symp.*, vol. 2017, pp. 1430–1439, 2017.

[36] F. Prasser, F. Kohlmayer, and K. Kuhn, “The Importance of Context: Risk-based De-identification of Biomedical Data,” *Methods Inf. Med.*, vol. 55, no. 04, pp. 347–355, 2016, doi: 10.3414/ME16-01-0012.
[37] E. F. Webdev, “Eclipse Nebula - Supplemental Widgets for SWT,” *projects.eclipse.org*, Jan. 31, 2013. https://projects.eclipse.org/projects/technology.nebula (accessed Jul. 07, 2020).
[38] P. Samarati and L. Sweeney, “Generalizing data to provide anonymity when disclosing information (abstract),” in *Proceedings of the seventeenth ACM SIGACT-SIGMOD-SIGART symposium on Principles of database systems - PODS ’98*, Seattle, Washington, United States, 1998, p. 188, doi: 10.1145/275487.275508.
[39] *Sharing Clinical Trial Data: Maximizing Benefits, Minimizing Risk (Appendix B Concepts and Methods for De-identifying Clinical Trial Data)*. Washington, D.C.: National Academies Press, 2015, p. 18998.
[40] J. Pitman, “Random discrete distributions invariant under size-biased permutation,” *Adv. Appl. Probab.*, vol. 28, no. 2, pp. 525–539, Jun. 1996, doi: 10.2307/1428070.
[41] N. Hoshino, “Applying Pitman’s sampling formula to microdata disclosure risk assessment,” *J. Off. Stat.*, vol. 17, no. 4, p. 499, 2001.
[42] V. S. Iyengar, “Transforming data to satisfy privacy constraints,” in *Proceedings of the eighth ACM SIGKDD international conference on Knowledge discovery and data mining - KDD ’02*, Edmonton, Alberta, Canada, 2002, p. 279, doi: 10.1145/775047.775089.
[43] “UCI Machine Learning Repository: Adult Data Set.” http://archive.ics.uci.edu/ml/datasets/adult (accessed Jul. 07, 2020).
[44] “ATUS-X : ATUS Data Extract Builder.” https://www.atusdata.org/atus/index.shtml (accessed Jul. 07, 2020).
[45] “IPUMS NHIS.” https://nhis.ipums.org/nhis/ (accessed Jul. 07, 2020).
[46] U. C. Bureau, “American Community Survey (ACS),” *The United States Census Bureau*. https://www.census.gov/programs-surveys/acs (accessed Jul. 07, 2020).
[47] “UCI Machine Learning Repository: default of credit card clients Data Set.” https://archive.ics.uci.edu/ml/datasets/default+of+credit+card+clients (accessed Jul. 07, 2020).
[48] “Open psychology data: Raw data from online personality tests.” https://openpsychometrics.org/_rawdata/ (accessed Jul. 07, 2020).
[49] A. Solanas, A. Martinez-Balleste, J. M. Mateo-Sanz, and J. Domingo-Ferrer, “Multivariate Microaggregation Based Genetic Algorithms,” in *2006 3rd International IEEE Conference Intelligent Systems*, London, UK, Sep. 2006, pp. 65–70, doi: 10.1109/IS.2006.348395.
[50] J. Casas-Roma, J. Herrera-Joancomartí, and V. Torra, “Comparing Random-Based and k-Anonymity-Based Algorithms for Graph Anonymization,” in *Modeling Decisions for Artificial Intelligence*, vol. 7647, V. Torra, Y. Narukawa, B. López, and M. Villaret, Eds. Berlin, Heidelberg: Springer Berlin Heidelberg, 2012, pp. 197–209.
[51] Y. He and J. F. Naughton, “Anonymization of set-valued data via top-down, local generalization,” *Proc. VLDB Endow.*, vol. 2, no. 1, pp. 934–945, Aug. 2009, doi: 10.14778/1687627.1687733.
[52] B. C. M. Fung, Ke Wang, and P. S. Yu, “Top-Down Specialization for Information and Privacy Preservation,” in *21st International Conference on Data Engineering (ICDE’05)*, Tokyo, Japan, 2005, pp. 205–216, doi: 10.1109/ICDE.2005.143.
[53] B. C. M. Fung, K. Wang, R. Chen, and P. S. Yu, “Privacy-preserving data publishing: A survey of recent developments,” *ACM Comput. Surv.*, vol. 42, no. 4, pp. 1–53, Jun. 2010, doi: 10.1145/1749603.1749605.
[54] T. K. Nayak and S. A. Adeshiyan, “On Invariant Post-randomization for Statistical Disclosure Control: Invariant PRAM for Disclosure Control,” *Int. Stat. Rev.*, vol. 84, no. 1, pp. 26–42, Apr. 2016, doi: 10.1111/insr.12092.

Figure1 Click here to access/download;Figure;Figure1.png

Age	Sex	ZIP	BMI	Diagnosis
55	M	92855	28.0	I47.1 Supraventricular tachycardia
91	M	94552	24.9	I50.1 Left ventricular failure
85	F	92855	26.7	I50.0 Congestive heart failure
68	F	94230	31.7	I47.1 Supraventricular tachycardia
77	F	92855	18.3	I47.0 Re-entry ventricular arrhythmia
82	F	92855	24.0	I50.0 Congestive heart failure
78	F	92301	28.1	I50.0 Left ventricular failure
75	M	94550	31.0	I47.0 Re-entry ventricular arrhythmia
82	M	94501	34.9	I47.0 Re-entry ventricular arrhythmia
69	M	93321	32.3	I50.1 Left ventricular failure

1 2 3 4 5 6

Age	Sex	ZIP	BMI	Diagnosis
81.7	F	92***	19-29	I50 Heart failure
81.7	F	92***	19-29	I50 Heart failure
81.7	F	92***	19-29	I50 Heart failure
75.0	*	94***	≥ 30	I47 Paroxysmal tachycardia
75.0	*	94***	≥ 30	I47 Paroxysmal tachycardia
75.0	*	94***	≥ 30	I47 Paroxysmal tachycardia

1 Sampling
2 Aggregation
3 Supression
4 Masking
5 Categorization
6 Generalization

Figure2 Click here to access/download;Figure;Figure2.png

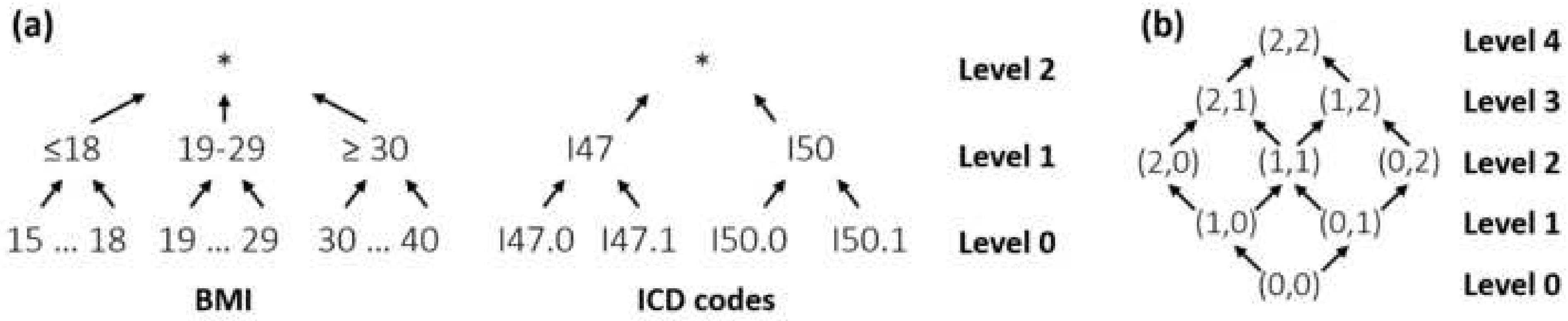

Figure3

Click here to access/download;Figure;Figure3.PNG

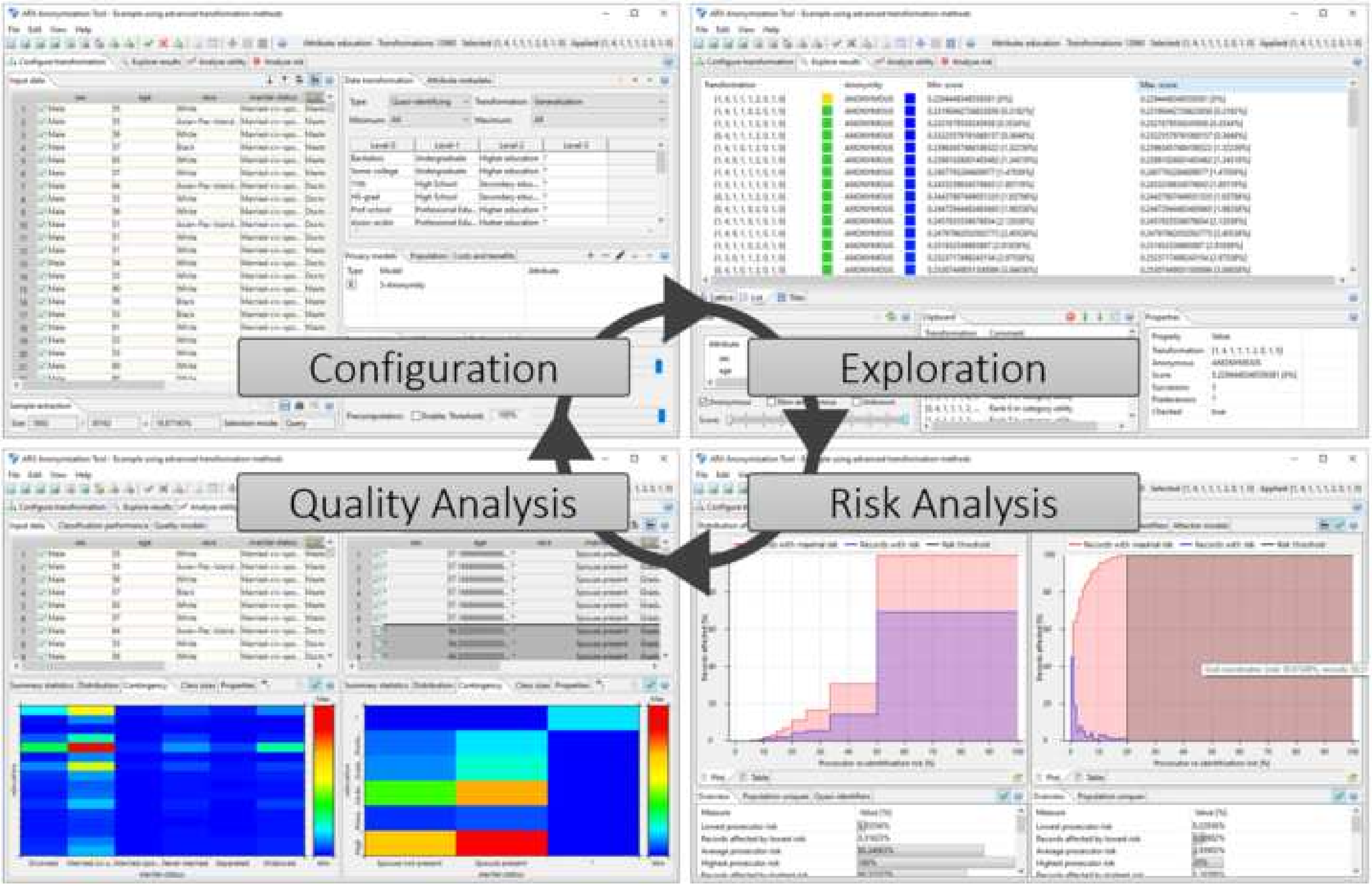

Figure4 Click here to access/download;Figure;Figure4.PNG

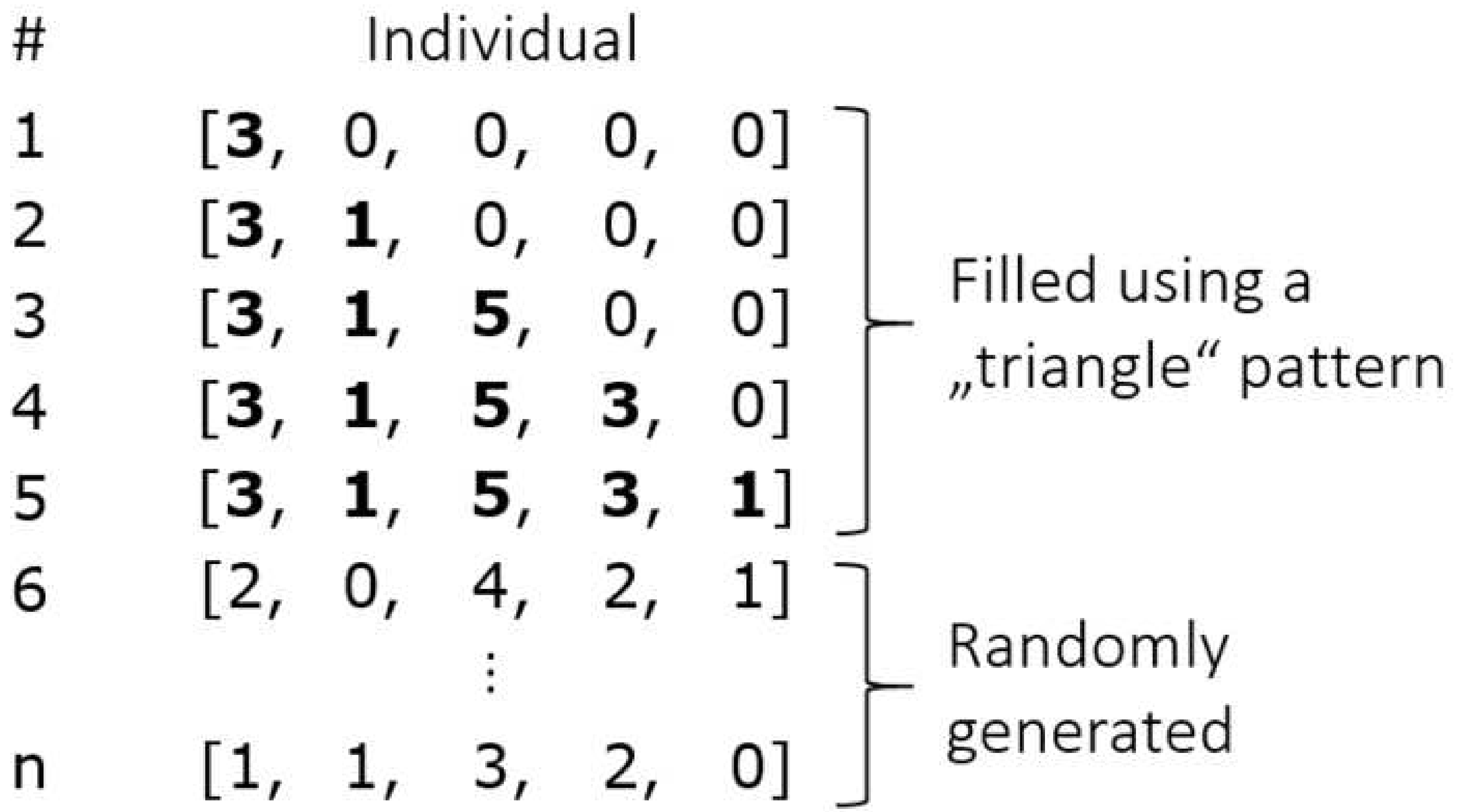

Figure5

Click here to access/download;Figure;Figure5.PNG

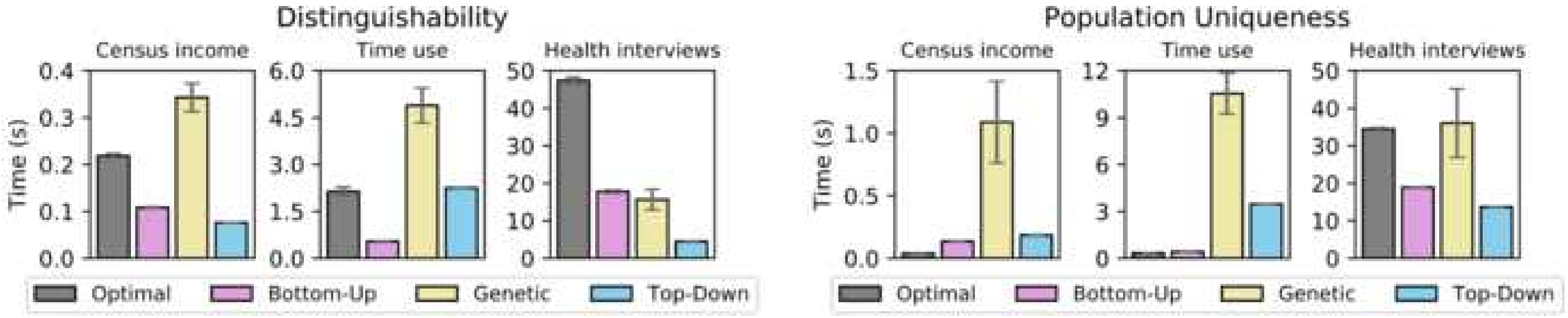

Figure6

Click here to access/download;Figure;Figure6.PNG

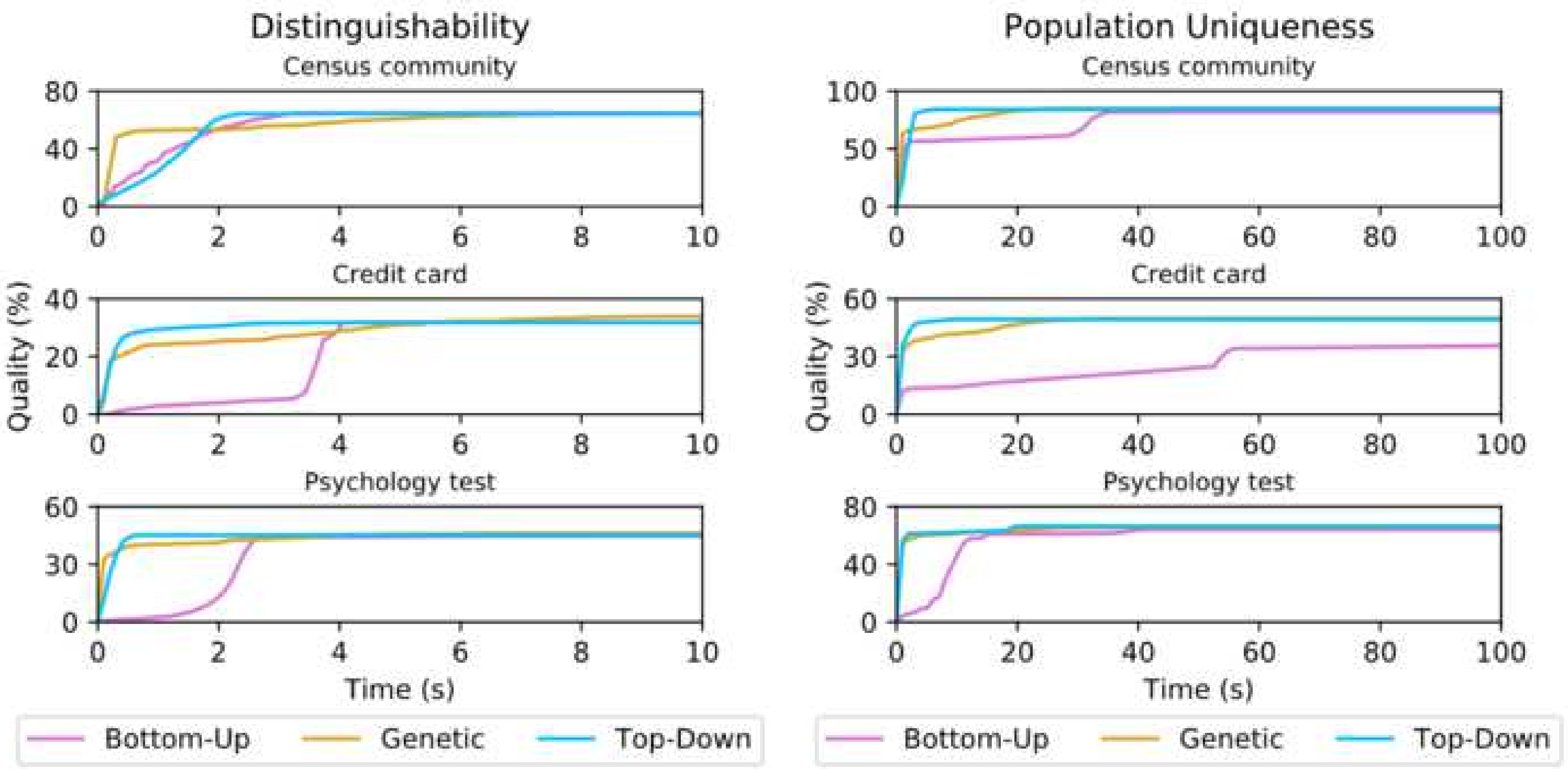

Figure7

Click here to access/download;Figure;Figure7.PNG

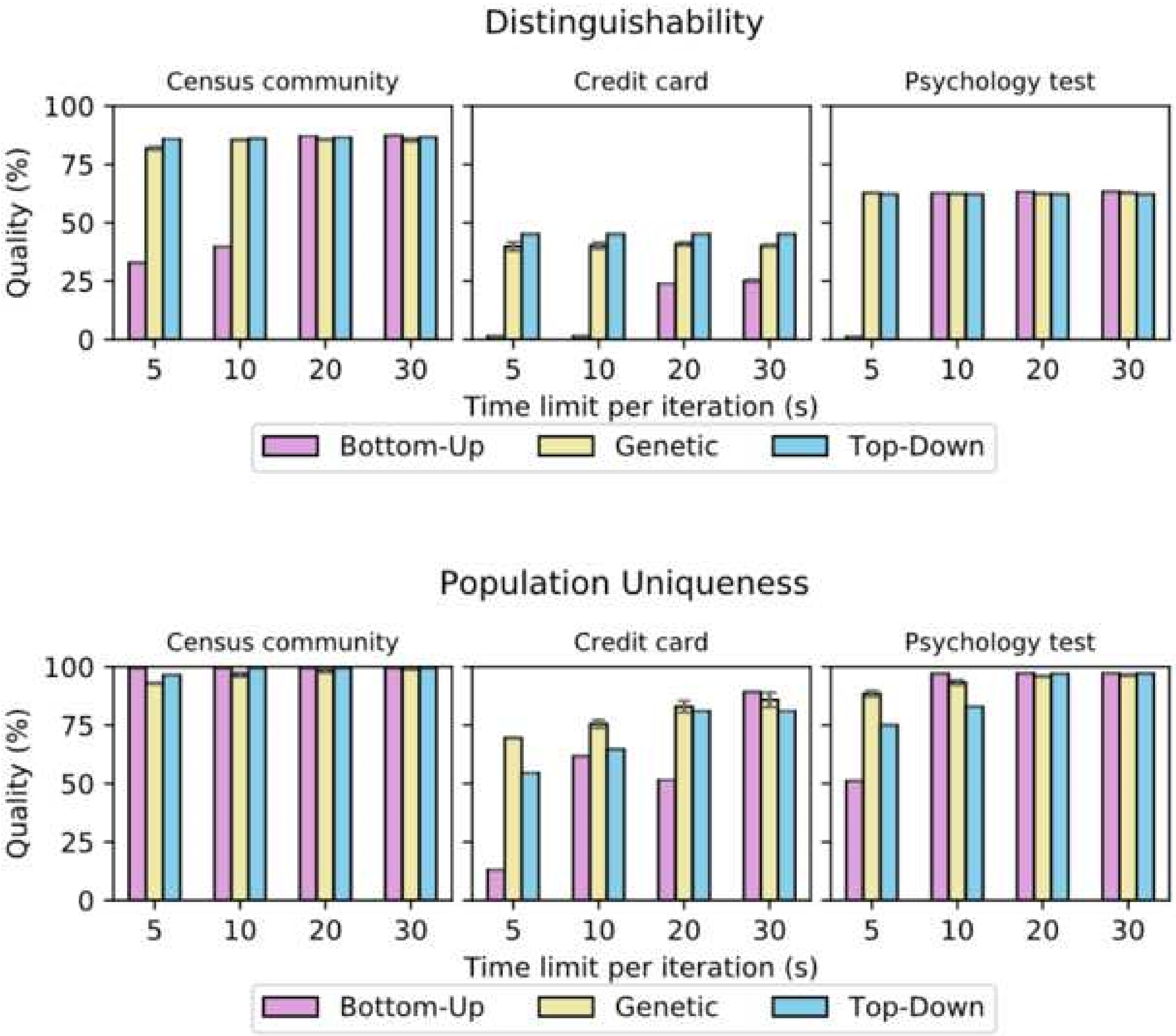

Figure8

Click here to access/download;Figure;Figure8.PNG

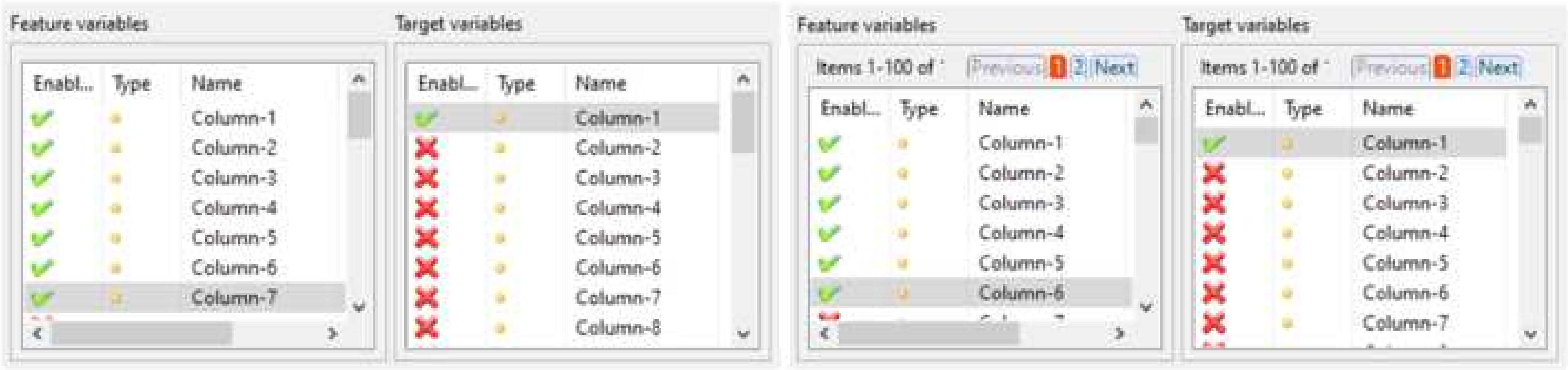

Supplement: giab068_GIGA-D-20-00292_Original_Submission [file giab068_giga-d-20-00292_original_submission.pdf]
