# Supplementary material for: A scalable software solution for anonymizing high-dimensional biomedical data
Source: Gigascience. 2021 Oct 4;10(10):giab068. doi: 10.1093/gigascience/giab068 (PMC8489190; doi:10.1093/gigascience/giab068)
Supplement: giab068_GIGA-D-20-00292_Revision_2 [file giab068_giga-d-20-00292_revision_2.pdf]

GigaScience

A scalable software solution for anonymizing high-dimensional biomedical data

--Manuscript Draft--

Manuscript Number:	GIGA-D-20-00292R2
Full Title:	A scalable software solution for anonymizing high-dimensional biomedical data
Article Type:	Technical Note
Funding Information:	
Abstract:	Background: Data anonymization is an important building block for ensuring privacy and fosters the re-use of data. However, transforming the data in a way it preserves the privacy of subjects while maintaining a high degree of data quality is challenging and particularly difficult when processing complex datasets that contain a high number of attributes. In this paper we present how we extended the open source software ARX to improve its support for high-dimensional, biomedical datasets.<br><br>Findings: For improving ARX's capability to find optimal transformations when processing high-dimensional data, we implement two novel search algorithms. The first one is a greedy top-down approach and is oriented on a formally implemented bottom-up search. The second is based on a genetic algorithm. We evaluated the algorithms with different datasets, transformation methods and privacy models. The novel algorithms mostly outperformed the previously implemented bottom-up search. Additionally, we extended the graphical user interface to provide a high degree auf usability and performance when working with high-dimensional datasets.<br><br>Conclusion: With our additions we have significantly enhanced ARX's ability to handle high-dimensional data in terms of processing performance as well as usability and thus can further facilitate data sharing.
Corresponding Author:	Thierry Meurers<br>Charite Universitatsmedizin Berlin<br>Berlin, GERMANY
Corresponding Author Secondary Information:	
Corresponding Author's Institution:	Charite Universitatsmedizin Berlin
Corresponding Author's Secondary Institution:	
First Author:	Thierry Meurers
First Author Secondary Information:	
Order of Authors:	Thierry Meurers
	Raffael Bild
	Kieu-Mi Do
	Fabian Prasser
Order of Authors Secondary Information:	
Response to Reviewers:	--- Reviewer 1 ---:<br>Comment 1: The manuscript has significantly improved.<br><br>Response: We thank the reviewer for this kind feedback! The remaining comments are addressed below.<br><br>Comment 2: Meanwhile, it would be better to restructure the contents. For example, all figures at the end are not desirable, and are hard to understand.<br><br>Response: We thanks the reviewer for pointing this out. We carefully revised the structure of the document and decided to move Figure 4, to include a more thorough

Powered by Editorial Manager® and ProduXion Manager® from Aries Systems Corporation

description of the figure and to move Section 2.5.3 (“Datasets”) as well as the table within this section. We would like to point out that all figures were uploaded separately to comply with the journal’s submission guideline. This is the reason for them currently appearing at the end of the document. However, their final positions in the manuscript are indicated by placeholders and captions, so they can be moved to their correct position during typesetting.

Comment 3: Also, it would be better to discuss the details of high and low dimensional data.

Response: We thank the reviewer and agree that this needed further clarification. We revised Section 1 (“Introduction”) to describe more clearly what we mean by low- and high-dimensional and to emphasize the challenges related to the anonymization of high-dimensional data. Furthermore, we added more details about the dimensionality of the datasets used to Section 2.5.1 (“Experiments”).

Comment 4: Please modify the comprehensive figure to highlight the complete workflow of the approach with data.

Response: We thank you for this suggestion. To improve the illustration of our approach, we decided to revise two Figures (2 and 4) and to add additional explanations. Figure 2 now illustrates how an exemplary dataset can be transformed by applying different generalization schemes from the solution space. We also revised Figure 4 to be directly based on the example presented in Figure 2 to convey a more complete picture. We also revised and extended the explanation of the genetic algorithm in Section 2.3 (“Integrating Anonymization Algorithms for High-Dimensional Data”), which now relates to the example provided in Figure 4.

Comment 5: A final version should depict the good structure of the paper that can be perceived easily by the readers.

Response: We would like to thank the reviewer once again for the important comments regarding the structure. We have reviewed the structure thoroughly, made the changes described above and are optimistic that the reading flow has improved significantly (especially when all the illustrations are moved to their final position during typesetting).

--- Reviewer 2 ---:
Comment 1: The authors have responded appropriately to the issues raised, and the manuscript has been modified accordingly. There are no further queries.

Response: We thank the reviewer for this positive feedback and for the valuable comments made on the previous version of the manuscript!

--- Editor ---:
Comment 1: [The reviewers] have requested a final check of the formatting,

Response: As described in our response to the comments by reviewer 1, we have thoroughly checked the structure and formatting of the paper to improve its reading flow. We believe that the manuscript has improved significantly in this regard.

Comment 2: and we would also recommend you follow the software citation guidelines for all software mentioned in the paper:
https://academic.oup.com/gigascience/pages/technical_note

Response: Thank you for pointing this out. We checked all references to software and revised the citation of ARX and included a new to our benchmark code.

Comment 3: I've cc'd our curators here who will help you archive a snapshot of the code and any other materials in our GigaDB repository, giving it a DOI that you will need to cite in an Data Availability section in the paper.

Response: Thanks to the help of the curators, a snapshot of our supporting code and data was uploaded to a GigaDB repository. We added a reference for the DOI and included it in the Data Availability section.

Powered by Editorial Manager® and ProduXion Manager® from Aries Systems Corporation

Additional Information:	
Question	Response
Are you submitting this manuscript to a special series or article collection?	No
**Experimental design and statistics**<br><br>Full details of the experimental design and statistical methods used should be given in the Methods section, as detailed in our Minimum Standards Reporting Checklist. Information essential to interpreting the data presented should be made available in the figure legends.<br><br>Have you included all the information requested in your manuscript?	Yes
**Resources**<br><br>A description of all resources used, including antibodies, cell lines, animals and software tools, with enough information to allow them to be uniquely identified, should be included in the Methods section. Authors are strongly encouraged to cite Research Resource Identifiers (RRIDs) for antibodies, model organisms and tools, where possible.<br><br>Have you included the information requested as detailed in our Minimum Standards Reporting Checklist?	Yes
**Availability of data and materials**<br><br>All datasets and code on which the conclusions of the paper rely must be either included in your submission or deposited in publicly available repositories (where available and ethically appropriate), referencing such data using a unique identifier in the references and in the "Availability of Data and Materials" section of your manuscript.	Yes

Powered by Editorial Manager® and ProduXion Manager® from Aries Systems Corporation

Have you have met the above requirement as detailed in our Minimum Standards Reporting Checklist?	

Manuscript

Click here to access/download;Manuscript;Manuscript_rev2_f1.docx

A scalable software solution for anonymizing high-dimensional biomedical data

Thierry Meurers[1] [0000-0001-8168-7067] (thierry.meurers@charite.de) (corresponding Author),

Raffael Bild[2] [0000-0002-7398-5598] (raffael.bild@tum.de),

Kieu-Mi Do[3] (kieumi.do@tum.de),

Fabian Prasser[1] [0000-0003-3172-3095] (fabian.prasser@charite.de)

1 Berlin Institute of Health at Charité – Universitätsmedizin Berlin, Charitéplatz 1, 10117 Berlin, Germany

2 School of Medicine, Technical University of Munich, Ismaninger Str. 22, 81675 Munich, Germany

3 Faculty of Informatics, Technical University of Munich, Boltzmannstr. 3 85748 Garching, Germany

Abstract

Background: Data anonymization is an important building block for ensuring privacy and fosters the re-use of data. However, transforming the data in a way it preserves the privacy of subjects while maintaining a high degree of data quality is challenging and particularly difficult when processing complex datasets that contain a high number of attributes. In this paper we present how we extended the open source software ARX to improve its support for high-dimensional, biomedical datasets.

Findings: For improving ARX's capability to find optimal transformations when processing high-dimensional data, we implement two novel search algorithms. The first one is a greedy top-down approach and is oriented on a formally implemented bottom-up search. The second is based on a genetic algorithm. We evaluated the algorithms with different datasets, transformation methods and privacy models. The novel algorithms mostly outperformed the previously implemented bottom-up search. Additionally, we extended the graphical user interface to provide a high degree auf usability and performance when working with high-dimensional datasets.

Conclusion: With our additions we have significantly enhanced ARX's ability to handle high-dimensional data in terms of processing performance as well as usability and thus can further facilitate data sharing.

Keywords

data privacy, anonymization, de-identification, heuristics, genetic algorithm, software tool, privacy preserving data publishing, biomedical data, data protection

1 Introduction

Big data technologies and latest data science methods promise to be valuable tools for providing new insights into the development and course of diseases. These insights can be used to derive new preventive, diagnostic and therapeutic measures [1]. Implementing these methods in practice requires

access to comprehensive, multi-level datasets of high quality. At a large scale, this can only be achieved by fostering the reuse of data from different contexts and the sharing of data across institutional boundaries. The reuse of data is also in line with the FAIR (Findable, Accessible, Interoperable, Reusable) data principles and supports the reproducibility of research. However, in the context of biomedical research, sharing data is challenging as it is important to account for ethical aspects [2], privacy concerns as well as data protection laws like for example the US Health Insurance Portability and Accountability Act (HIPAA) [3] or the European General Data Protection Regulation (GDPR) [4].

One important building block for ensuring privacy is to provide safe data that minimizes disclosure risks [5]. This can be achieved by employing data anonymization techniques, that transform the data to mitigate privacy risks [6], [7]. Typically, the anonymization process is not limited to the removal of directly identifying attributes such as the name, telephone number or insurance id number. Instead, it must also account for attributes like the postal code, age and gender that could be combined to re-identify individuals or derive sensitive personal information [8]–[10]. However, transforming the data will also have an impact on its usefulness and striking the right balance between privacy and data quality is challenging. The complexity of this task is also demonstrated by several re-identification attacks [11], [12]. As most anonymization approaches are based on the idea of reducing the uniqueness of attribute combinations, preserving a reasonable amount of information becomes particularly difficult when working with high-dimensional datasets that contain a high number of attributes [13]. Furthermore, the number of possible transformations of a dataset usually grows exponentially with the number of its attributes, leading to computational challenges [14]. Thus, the literature mostly addresses the anonymization of low-dimensional datasets featuring up to 10 or 15 attributes [15]–[18].To put anonymization of high-dimensional datasets into practice, tools that support a variety of mathematical and statistical privacy models and allow for their combination must feature scalable algorithms capable of approximating suitable solutions. An example of such a tool is the open source software ARX [6], [19]. It is focused on biomedical data and has been mentioned in several official

policies and guidelines [20], [21], used in research projects [22]–[24], and enabled several data publishing activities [25]–[27].

Versions of ARX up to 3.8.0 were only able to process datasets with a limited number of attributes that could be considered during anonymization (up to about 15). The reason for this were twofold: (1) the software only had limited support for anonymization algorithms able to process high-dimensional data, (2) the graphical user interface was not designed to work with datasets containing a high number of attributes.

In this Technical Note, we describe our efforts to overcome these limitations by (1) extending ARX's user interface with additional views that simplify the management of high-dimensional data, (2) implementing two novel heuristic anonymization algorithms and (3) evaluating the novel algorithms regarding their performance for anonymizing low-dimensional and high-dimensional datasets.

2 Materials and Methods

In this section, we will first provide some fundamental details about data anonymization. Second, we will present important properties of the ARX Anonymization Tool that had an influence on our design decisions. Third, we will present the extensions implemented into ARX. Finally, we will provide insights into our experimental setup.

2.1 Fundamentals of Data Anonymization

When anonymizing a dataset the first step is to remove all attributes that directly identify the individuals. Thereafter, the dataset is modified or noise is introduced so that the risk of identified or identifiable individuals of being linked to one or multiple records of the dataset or to sensitive information in general is lowered [7]. This step involves the usage of mathematical or statistical privacy models used to quantify the risk of privacy breaches as well as quality models that measure the usefulness of the output data. For (1) measuring privacy risks, (2) measuring data quality and (3) transforming the data a variety of models can be employed and combined.

< Figure 1 >

Figure 1: Exemplary anonymization process.

Figure 1 shows a simplified example of an anonymization process. The transformation involves different procedures such as (1) randomly sampling the records, (2) aggregating values by replacing them with their mean, (3) suppressing values, (4) masking trailing characters of strings, (5) categorizing numerical values and (6) generalizing categorical attributes. These transformations may reduce the fidelity of the data but also reduce the risk of linkage attacks and the attacker's accuracy when linking records. Furthermore, an additional uncertainty could be created by introducing noise. The transformed output data of the example fulfills two frequently used privacy models: k-Anonymity with $k = 3$ [28] and (ε, δ)-Differential Privacy with $\varepsilon \approx 0.92$ and $\delta \approx 0.22$ [29].

The simple example demonstrates the variety of possibilities available for transforming data. Furthermore, it also suggests why it is often not feasible to search the entire solution space of all potential output datasets when processing more complex data. For this kind of tasks, solutions that try to determine a good transformation scheme on a best-effort basis, e.g. based on heuristic strategies [15], [30], [31] or clustering algorithms [16], [17], [32], have been developed. An overview of common types of approaches is provided by Fung et al. [7].

2.2 The ARX Anonymization Tool

ARX supports a variety of privacy models, quality models and data transformation schemes and allows for their arbitrary combination [6]. For transforming the data, it relies on domain generalization hierarchies which describe how values can be transformed to make them less unique. For each hierarchy it is possible to define multiple levels of generalization that cover an increasing range of the attribute's domain. The basic solution space that is utilized by ARX is given by all possible combinations of generalization levels defined by the hierarchies. These combinations are referred to as generalization schemes.

< Figure 2 >

Figure 2: Generalization hierarchies (a) and the structure of the corresponding solution space together with examples of how data is transformed when applying different generalization schemes using global generalization(b).

Figure 2(a) shows exemplary generalization hierarchies for the attributes BMI, Sex and ICD. Figure 2(b) illustrates how the solution space resulting from these hierarchies is structured and how applying different generalization schemes would alter an exemplary dataset.

Mathematically, the solution space is a lattice [33], [34], which grows exponentially in size regarding the number of attributes that need to be protected [31]. As ARX is also able to apply different generalization schemes automatically to different parts of the input dataset the size of the solution space may grow further by a multiplicative factor representing the number of rows [6]. ARX supports different algorithms for finding optimal solutions within solution spaces of tractable size [35] as well as a heuristic algorithm for larger search spaces that tries to determine a good transformation scheme on a best-effort basis [31].

In addition to its anonymization engine, ARX also features a cross-platform graphical user interface. An overview of the different perspectives provided by the platform is shown in Figure 3.

< Figure 3 >

Figure 3: Basic perspectives of the graphical interface of the ARX Data Anonymization Tool.

In the *configuration* perspective it is possible to define risk thresholds for different types of attacks, to prioritize attributes by importance, to model the background knowledge of possible attackers and to define transformation methods and rules. In the *exploration* perspective, relevant anonymization strategies are visualized for the input data and a categorization according to output data quality is supported. A further perspective supports the manual *quality analysis* of the output data. Different methods for measuring the information content of the output data, descriptive statistics and methods for comparing the usefulness of the input and output data for different application scenarios are provided. In a *risk analysis* perspective, it is possible to visually compare input and output data using

different risk models. However, in the user interface it is challenging to support high-dimensional datasets. For example, several perspectives and views of the software display lists of all attributes of the dataset loaded, which can become confusing and lead to performance problems on some platforms with an increasing number of attributes.

2.3 Integrating Anonymization Algorithms for High-Dimensional Data

As mentioned before, the anonymization procedures supported by ARX are built around a basic operator that searches through the generalization lattice. In prior work we have already integrated a greedy best-first bottom-up search algorithm into the software [31]. This algorithm starts at the bottom generalization scheme, which applies no generalization to the data. It then "expands" this generalization scheme, by applying all generalization schemes to the input dataset that can be derived by increasing one of the generalization levels. The quality of the resulting output dataset is computed for all these schemes, and the process is repeated by expanding the generalization resulting in the dataset with highest quality. This process is then repeated until a user-specified period of time has passed. During the execution of the algorithm, a list of all generalization schemes that have been evaluated is stored and, in each iteration, the scheme with the highest output data quality that has not yet been expanded is expanded. For further details we refer interesting readers to the original publication [31].

It must be noted that this process is only suitable for processing dataset of medium dimensionality (about 15 attributes) for several reasons. First, the search process may become trapped in local minima, as there is no significant diversification of the solutions considered. Second, the process naturally favors transformation schemes located in the lower part of the search space (i.e. schemes that apply a low degree of generalization). While this makes sense for anonymization processes that only apply generalization, the method reaches its limits with the complex transformation operations supported in newer versions of ARX in which different transformation schemas are used to transform different parts of a dataset. In this case, a better overall solution can sometimes be determined if

outliers are transformed more strongly. To further improve this process, we have integrated two new algorithms for processing high-dimensional data into the software.

The first algorithm closely resembles the bottom-up greedy best-first search but performs this process top-down. We will not describe it in further detail, as this is a straight-forward extension of the process described in the previous paragraphs.

The second algorithm applies a genetic optimization process to the anonymization problem. Genetic algorithms search for solutions in a heuristic manner that is oriented on the process of natural selection [36]. During the search, the solutions are considered individuals that carry the solution's properties encoded as a list of genes (in our application, the individuals carry generalization schemes and genes correspond to the generalization level of specific attributes). The set of candidate solutions/individuals is called population. Mostly, the initial population is created by randomly generating individuals. Thereafter, the algorithm works iteratively. By crossing (i.e. randomly combining the properties of two individuals) and mutating (i.e. randomly altering the properties of individuals) selected individuals contained in the population, each iteration will result in a new, so-called, generation. Whether and how an individual is altered is determined by its fitness which usually is calculated using the cost function of the investigated optimization problem. Once reaching a predefined limit of iterations the fittest individual is considered the optimal solution. However, there is no guarantee that a globally optimal solution can always be found.

We opted for the genetic algorithm as it is one of the most well-known population-based meta-heuristics. In comparison to single-solution based algorithms (e.g. simulated annealing or the greedy heuristics described above) population-based approaches maintain multiple candidate solutions which potentially results in a high degree of diversification and a decreased risk of getting stuck in local optima [37]. Moreover, genetic algorithms have already been successfully applied for anonymizing data in previous work. However, prior approaches were often limited to a specific kind of data or privacy model (see Section 4.3 "Comparison with Prior Work"). The genetic algorithm implemented

into ARX is based on the work by Wan et al. [38]. Wan et al. employed the algorithm for anonymization genomic data using a game-theoretic privacy model, which was already successfully adapted and integrated into ARX in prior work [39].

Figure 4 illustrates how the algorithms search through the solution space to find a good generalization scheme, based on the example presented in Figure 2. Although the process shown in the figure is simplified, it illustrates that both approaches follow completely different concepts.

< Figure 4 >

Figure 4: Illustration of (a) the top-down approach and (b) the genetic algorithm searching the solution space. Generalization schemes visited that were not on the path to the best solution are colored grey. The best scheme found is marked by a double border.

To make the genetic algorithm compatible with the types of solution spaces used by ARX and to integrate it with the privacy and quality models supported by the software every individual carries a list of numerical values representing a generalization scheme. The list's length (i.e. the number of genes) equals the number of attributes that need to be transformed and the i-th value of the list represents the generalization level of the i-th attribute. The range of each value is given by the lowest and highest generalization level available for the corresponding attribute. Applied to the example illustrated in Figure 4, this results in individuals carrying three genes with values between 0 and 2. Implementation-wise the populations are maintained in a matrix like structure with the rows of this matrix representing individuals (generalization scheme) and columns their genes (generalization level of an attribute). ARX's privacy and quality models have been integrated via the fitness function. ARX always automatically alters the output of any given transformation in such a way that the required privacy guarantees are provided. This is achieved by suppressing records [40]. The suppression of records is captured by a decrease in data quality. Hence, we defined the fitness of a transformation to

equal output data quality, which not only measures the transformation's direct impact on data quality but also implicitly captures how well the required privacy guarantees are achieved.

The algorithm itself works as follows:

- **Initialization:** During the initialization two equally sized subpopulations are created. Following the approach of Wan et al., the first individuals of the first subpopulation are generated in the form of a "triangle pattern" using the lowest and highest generalization levels. An example is provided in Figure 5. The remaining individuals of the first subpopulation as well as the entire second subpopulation is filled by randomly creating individuals. The motivation behind this approach is based on properties of genetic data [41]. To determine whether the initialization procedure is also favorable in our case, we performed experiments in which we compared the initialization strategy proposed by Wan et al. with a completely random initialization in a single- as well as a dual-population setting (see Supplementary Table S1). The experiments showed that using the "triangle pattern" performs well when processing low-dimensional data and significantly outperforms other approaches when processing high-dimensional datasets. This can be explained by the fact that the pattern creates populations that cover a larger part of the solution space in the beginning.

< Figure 5 >

Figure 5: Initialization of the first subpopulation for a solution space with the highest generalization levels of [3,1,5,3,1].

- **Iteration:** After initializing the subpopulations the algorithm's main loop is started. The algorithm stops after reaching a pre-defined number of iterations or time limit. Within the loop the following steps are executed:

  Step 1: Sorting: The individuals contained in the subpopulations are sorted by their fitness in descending order.

  Step 2: Selection: The fittest individuals of the current population will simply be copied to the next generation without being modified. We refer to this fraction of individuals as *elite*

fraction. In Figure 4(b), this mechanism is indicated by an arrow, with which an individual points to itself since it remains unchanged.

Step 3: Crossover: Next, the so-called *crossover fraction* of the new generation is populated. For this purpose, two parent-individuals from the *production fraction* of the current population are crossed to generate a new child-individual. The probability of being chosen as a parent increases with the fitness. The crossover is performed in a randomized fashion. For every gene it is decided randomly from which of the two parents it is inherited. Figure 4(b) illustrated how a child-individual inherits the genes of its ancestors. Which gene was inherited from which parent can be distinguished by the color coding.

Step 4: Mutation: The rest of the new generation is populated by randomly choosing individuals of the current generation and mutating them by altering their genes. The number of changed genes is randomly chosen between 1 and an upper bound which is calculated by multiplying the *mutation probabilit*y with the number of available genes. Figure 4(b) depicts an individual which is being mutated at one of its genes while leaving the remaining genes unchanged. The mutated gene is indicated by a change in color.

Step 5: Swapping: Additionally, it is possible that the fittest individuals are swapped between the two subpopulations. How often they are changed depends on the *immigration interval* which refers to the number of iterations between the swaps. The number of exchanged individuals can be controlled by the *immigration fraction*.

2.4 Extending the User-Interface for High-Dimensional Data

ARX is implemented as a cross-platform program using Java and executed on the Java Virtual Machine. The Graphical User Interface (GUI) is implemented using the Standard Widget Toolkit (SWT), which enables implementing native GUIs on three supported platforms: Windows, Linux and MacOS.

For improving the GUI's usability when working with high-dimensional datasets we made use of two SWT-based components provided by the Eclipse Nebula Project [42]. The first is NatTable. Based on

the idea of virtual tables it ensures that the GUI remains responsive and provides a high rendering performance when displaying large datasets. The second is Pagination Control. This component is used to display a navigation page when working with tables used to configure a potentially large number of attributes.

Additionally, ARX features a mechanism that automatically detects the type of an attribute to ease the initial import of data as well as the ability to configure multiple attributes at once. These last two features are also available for smaller dataset but are especially helpful when working with high-dimensional datasets.

2.5 Experimental Design

2.5.1 Experiments

With the extensions described in this article, ARX now supports three algorithms for anonymizing high-dimensional data: (1) the initial bottom-up search, (2) the new top-down search and (3) the new genetic search algorithm. We performed a series of experiments, to study how well these algorithms work for different types of data to provide users with insights into which algorithm should be used in which context. For the experiments we used low-dimensional datasets with less than 10 attributes and high-dimensional datasets containing up to 30 attributes (more details about the datasets are provided in Section 2.5.3). Depending on the dimensionality of the datasets we conducted two types of experiments:

(1) **Experiments with low-dimensional data:** We compared the algorithms to the optimal algorithm already supported by ARX [35] in the low-dimensional setting. We did this for two reasons. First, heuristic algorithms might also be relevant when anonymizing low-dimensional data if they significantly outperform optimal algorithms in terms of the time needed to find the optimal solution. Second, experiments with low-dimensional data might provide insights into basic strengths and weaknesses of the approaches. To this end, we compared the overall

execution time of ARX's optimal algorithm with the time needed by the heuristic algorithms to find the optimal solution.

(2) **Experiments with high-dimensional data:** Here, we use the three heuristic algorithms to anonymize high-dimensional datasets. This experiment was performed to determine whether the novel approaches (genetic and top-down) offer an advantage over the bottom-up algorithm. To this end, we executed the algorithms with different time limits and compared the quality of their results.

2.5.2 Privacy, quality and transformation model

To investigate a broad spectrum of anonymization problems, we decided to utilize different privacy and data transformation models.

For measuring and managing privacy risks, we used two models:

(1) **Distinguishability:** To implement restrictions on the distinguishability of data, we utilized the well-known and relatively strict k-anonymity model. A dataset is k-anonymous if every record cannot be distinguished from at least $k - 1$ other records in respect to attributes that may be used to de-anonymize the data [43]. As a parameter we used $k = 5$ which is a common recommendation [44].

(2) **Population uniqueness:** ARX also supports statistical models that estimate disclosure risks by estimating the fraction of records in a dataset that are expected to be unique in the overall population. Compared to k-anonymity, this is a relatively weak privacy model. For our experiments we enforced a uniqueness of $1\ \%$ within the US population and relied on the model introduced by Pitman to estimate population characteristics [45], [46].

For transforming data, we also used two common models:

(1) **Global generalization**: With this model, the values in a dataset are generalized based on user-defined hierarchies. In this process, it is guaranteed that all values of an attribute are

generalization to the same level of the associated hierarchy. To prevent overgeneralization, records can also be removed from the dataset.

(2) **Local generalization:** With this model, data is also transformed by generalization, but values of the same attribute in different records can be transformed differently. Records may also be removed, but this is typically not required due to the flexibility of the transformation model.

In ARX, local transformations are implemented by using an iterative process in which the dataset is automatically partitioned and different transformation schemes are applied to different partitions [6]. In our experiments with local generalization, we used 100 iterations and different time limits for individual iterations.

To quantify data quality, we decided to use the intuitive "Granularity" model [47], which measures the value-level precision of the output data. The measurements are normalized with $0\ \%$ representing a dataset from which all information has been removed and $100\ \%$ corresponding to a completely unmodified dataset [6].

2.5.3 Datasets

For evaluating the performance of the heuristic algorithms, we used six different real-world datasets. An overview of the properties of the datasets is shown in Table 1.

Table 1: Overview of the datasets used for comparing the algorithms.

Name	#Attributes	#Records	Solution space size	Category
Census income [48]	9	30,162	12,960	Low dimensional
Time use [49]	9	539,254	34,992	Low dimensional
Health interviews [50]	9	1,185,424	25,920	Low dimensional
Census community [51]	30	68,725	203,843,174,400	High dimensional

Credit card [52]	24	30,000	49,478,023,249,920	High dimensional
Psychology test [53]	16	73,489	85,030,560	High dimensional

Most of the datasets have already been utilized in previous evaluations of data anonymization algorithms. As low-dimensional datasets we choose (1) an excerpt of the 1994 US census dataset (*Census income*) which can be considered the de-facto standard for evaluating anonymization algorithms, (2) data from a nationally representative U.S. time diary survey and (3) results from the integrated health interview series collecting data on the health of the U.S. population.

As high-dimensional datasets we included (1) data from the responses to the American Community Survey (ACS) which captures demographic, social and economic characteristics of people living in the U.S., (2) a credit card client dataset from Taiwan used to estimate costumers default payments and (3) answers to a psychological test designed to measure someone's Machiavellianism from the open-source psychometrics project. As attributes that needed to be transformed, we selected variables that are typically associated with a high risk of re-identification. These included demographic data, timestamps, spatial information, medical attributes and payment histories.

2.5.4 Parameterization

While the top-down and bottom-up search algorithms do not require any additional parameterization, the genetic search algorithm features multiple configuration parameters, which are shown in Table 2. In ARX, these parameters are presented as configuration options to the users.

Table 2: Parameters of the genetic algorithm and the values employed in the experiments.

Parameter	Description	Value
Elite fraction	Fraction of individuals that is directly copied to the next generation.	0.2

Crossover fraction	Fraction of individuals that is replaced by new individuals that are generated by crossing two parents from the production fraction.	0.4
Production fraction	Fraction of individuals used as parents when generating crossover individuals.	0.2
Mutation probability	Used to calculate the upper bound of changed genes when mutating individuals.	0.05
Immigration fraction	The fraction of individuals that is swapped between the subpopulation.	0.2
Immigration Interval	Number of iterations between swaps.	10
Iterations	Number of iterations performed by the GA	50
Subpopulation size	Number of individuals contained in each of the subpopulations.	50

Table 2 also shows the parameters used in our evaluation. Regarding all but one parameter we followed the suggested configuration by Wan et al. [38] for all parameters that are applicable to our setting. We made this decision based on a set of experiments performed in preparation of our evaluation in which we individually altered all parameters and examined their effect on the performance of the algorithm (see Supplementary Table S2). This process showed that setting the production fraction to 0.2 (instead of 0.8 as suggested by Wan et al.) improves execution times when processing low-dimensional datasets and data utility when processing high-dimensional datasets. The fact that almost the same parameters work well in our setting as well as in the experiments by Wan et al., although very different anonymization procedures are being investigated, can be seen as an indicator of the robustness and generality of this parameterization.

2.5.5 Technical Setup

We repeated each experiment five times and report the average for two reasons: first, it is well known that execution times of JVM-based programs vary slightly due to effects from functionalities, such as just-in-time compilation. Second, the genetic algorithm is randomized and hence may perform slightly different in each execution.

The experiments were performed on a desktop computer with an AMD Ryzen 2700X processor (8 cores, 3.7-4.3 GHz) running 64-bit Windows 10 (version 1909) and a 64-bit Oracle JVM (version 1.8.0).

3 Results

3.1 Experimental results

3.1.1 Low-dimensional data

The results of the first set of experiments are displayed in Figure 6. For each heuristic algorithm, it shows the time in seconds needed to determine the optimal solution (and the overall execution time for the optimal algorithm) using the global transformation model. We did not use the local transformation model in this experiment, as the underlying algorithm is heuristic in nature (independently of the actual search strategy used) and can therefore not be used to compare the time needed to achieve a specific result in terms of output data quality [6].

< Figure 6>

Figure 6: Time required for finding an optimal solution for different low-dimensional datasets and privacy models in dependence to the used algorithm.

As can be seen, heuristic approaches provided a valuable alternative to the optimal approach even in low-dimensional settings. When aiming for a threshold on distinguishability, the bottom-up and top-down search algorithms almost always outperformed the optimal algorithm. On average, the genetic algorithm was slower than the other heuristic approaches, because it aims at diversifying the solutions considered, which is not a desirable feature in low-dimensional settings. Whether the top-down approach or the bottom-up approach performed better was associated with the degree of generalization required and hence with the fact whether the optimal solution is located more closely to the top or to the bottom of the lattice.

When optimizing for a threshold on population uniqueness the optimal algorithm outperformed the heuristic approaches in two out of three cases. This can be explained by the fact that calculating population uniqueness is much more computationally complex than checking for k-anonymity, as bivariate non-linear equation systems need to be solved. As a consequence, execution times are not dominated by the time needed to transform the dataset but by the time needed to evaluate the privacy

model. The optimal approach implements a wide variety of pruning strategies that reduce the number of transformations that need to be checked [40], which cannot be implemented by the heuristic algorithms. The genetic algorithm provided the worst overall performance, as it tries to look at a diverse set of potential solutions.

3.1.2 High-dimensional data

The results of the experiments with high-dimensional data are displayed in Figure 7 and Figure 8. We compared the development of output data quality for the different algorithms over time and present two different types of results. For global transformation we continuously measured the development of output data quality over time. For local transformation we present the output data quality achieved with different time limits as the heuristic nature of the local transformation algorithm implemented in ARX makes it difficult to directly track the progress [6].

< Figure 7>

Figure 7: Global generalization: Quality improvement over time for different high-dimensional datasets and privacy models in dependence to the used algorithm.

Figure 7 shows the development of output data quality over time when using the global transformation model until the results of all three algorithms stabilized. As can be seen, all algorithms almost always eventually found a solution with comparable quality. However, when enforcing a threshold on population uniqueness on the credit card dataset, the bottom-up algorithm exhibited sub-optimal performance. Moreover, in most cases the genetic and top-down approach found better solutions much quicker than the bottom-up algorithm. When comparing the different algorithms to each other it can be seen that the genetic algorithm was generally good at quickly determining a relatively good solution while the top-down algorithm provided a good balance of optimization speed and quality of its overall output. It can also be seen that output data quality was higher when reducing population uniqueness compared to reducing distinguishability, as the former model is weaker than the latter (see Section 2.5.2).

Figure 8 provides additional insights by presenting the results for the local transformation model.

< Figure 8>

Figure 8: Local generalization: Achieved quality for different high-dimensional datasets depending on the privacy models and used algorithm.

Again, the time axis covers the time that was needed for the solutions of the different algorithms to stabilize. As can be seen, the results are quite similar to the results obtained using the global transformation model, apart from the fact that the overall output data quality is higher with this transformation method. The genetic algorithm is good at very quickly finding a relatively good transformation and in most cases all algorithms finally found a comparable solution. The credit card dataset is a notable exception. In this case, the bottom-up algorithm provided the best result when reducing population uniqueness and the top-down approach provided the best result when reducing distinguishability. It is notable that the genetic algorithm performed best for short time limits in the former case, as the credit card dataset results in the largest solution space and the evaluation of individual solution candidates is expensive for population uniqueness. Moreover, good solutions were not located close to the top or bottom of the search space. This is exactly the scenario in which one would expect good performance from a genetic search process.

3.2 Extended User-Interface

In the updated version of the ARX GUI, seven views of the software distributed over all four perspectives have been extended using the pagination feature. We note that this extension is graceful, meaning that it is only activated when a high-dimensional dataset is loaded into the software (an according threshold can be specified in the tool's settings). As an example, the pagination feature of a view in ARX's quality analysis perspective is shown in Figure 9.

< Figure 9>

Figure 9: Screenshots from the "Classification model" tab before (left) and after (right) adding the pagination feature.

Further features that are important for managing high-dimensional data with ARX, such as auto-detection of data types and options to configure multiple attributes at once, are located in different parts of the GUI, such as data import and hierarchy creation wizards as well as the software's main toolbar.

4 Discussion

4.1 Principal results

In this paper we presented the results of our efforts to improve the ability of the ARX Anonymization Tool to handle high-dimensional data. For this purpose, we extended the graphical user interface and introduced and evaluated two new heuristic anonymization algorithms. One of the algorithms, top-down search, complements the existing greedy bottom-up search algorithm. The other approach is based on a genetic algorithm and aims at diversifying the potential solutions considered using the process of natural selection.

Evaluating the newly implemented algorithms showed that they are particularly useful in scenarios where high-dimensional data needs to be anonymized. Using global generalization, they clearly outperformed the previously implemented bottom-up search (i.e. better performance in 5 of the 6 experiments). A similar result was observed when using local generalization. Averaged over all experiments, the new algorithms achieved a utility of 76.5 % (genetic algorithm) and 75.1 % (top-down algorithm), which is significantly higher than that provided by the bottom-up approach (60.2 %). Especially when anonymizing the dataset with the largest solution space (credit card), the new algorithms often performed significantly better, both in terms of scalability and utility. Additionally, the results obtained when processing low-dimensional data showed that heuristic algorithms can be helpful to improve computational efficiency even in scenarios where optimal algorithms could be used. The top-down approach required the least amount of time on average to find an optimal solution (4.0 s), followed by the bottom-up approach (6.3 s), the genetic algorithm (9.9 s) and the optimal search strategy (14.1 s).

Making a general recommendation for one of the algorithms is difficult based on the results of our experiments. For helping users to decide on an algorithm, ARX automatically determines whether it is feasible to calculate an optimal solution or whether a heuristic algorithm should be used. Also, ARX provides means to easily try out different algorithms and compare their results to enable users to determine which approach works best in which specific context.

4.2 Limitations

Our result show that the performance of the algorithms studied strongly depends on the dataset anonymized and the configuration utilized. While the new heuristic algorithms typically exhibited significantly improved performance in comparison to the methods previously implemented in ARX, this is not guaranteed to always be the case.

The exact operations of the genetic algorithm can be optimized by adjusting its parameterization. In our experiments, we used the parameterization by Wan et al. and additionally tuned the parameters for optimal average performance. Therefore, we chose a single parameterization in all our experiments. Optimizing the parameters for specific use cases could therefore lead to further improvements. For this reason, the GUI and API of ARX allow the user to easily change the parameterization of the genetic algorithm.

4.3 Comparison with Prior Work

It has been demonstrated multiple times that genetic algorithms can be used for anonymizing data. However, previously described solutions were mostly tailored towards specific types of data or privacy and transformation models.

Examples of approaches which focus on a specific type of data include the algorithm by Wan et al. [38], which targets genomic data and which we have adopted to general tabular data in this work, and the approach for anonymizing graphs presented by Casas-Roma et al. [54].

Regarding specific privacy and transformation models, genetic algorithms have also been used in clustering-based anonymization processes. To reduce distinguishability, such algorithms partition the

records of a dataset into several groups with each of the groups containing at least k members, hence implementing the k-anonymity model. Solanas et al. [55] demonstrated how the computationally challenging partition step which aims at maximizing homogeneity within the groups can be performed using a genetic algorithm. In their approach, the number of genes equals the number of records in the dataset with the i-th gene representing the group of the i-th record. The groups are encoded as an alphabet with a fixed size as the maximal number of different groups can be derived from k and the number of records in the dataset. Lin et al. [32] described how the scalability of the clustering process can be improved for large datasets by encoding the solution using the entire population instead of a single individual. Finally, focusing on data transformations, Iyengar [47] has demonstrated how a genetic algorithm can be used to determine intervals for generalizing values. In simplified terms each individual is a binary string with a length derived from the number of processed attributes and the number of their distinct values. A value of 1 in the string implies that a value is used as an interval boundary.

Our work is different from these approaches, because it integrates a genetic algorithm into ARX in such a way that it can be used to anonymize datasets using a variety of privacy models, quality models and data transformation schemes.

Heuristics anonymization algorithms comparable to the bottom-up approach evaluated in our paper include DataFly [30] and iGreedy [15]. Both use global generalization and are focused on k-anonymity only. They are based on a bottom-up search and follow the concept of minimal anonymization meaning they terminate as soon as they find a transformation that fulfills the requested privacy properties. In previous work we have already shown that the bottom-up algorithm implemented by ARX outperforms these approaches [31]. Furthermore, other researchers have focused on top-down search strategies. Important examples include the work of He et al. [56] who proposed a greedy top-down algorithm to partition a dataset and apply local generalization as well as the Top-Down Specialization method described by Fung et al. that iteratively specializes attributes until violating the anonymity requirements [57].

5 Conclusion and future work

With the work presented in this article we have significantly enhanced ARX's ability to handle high-dimensional data, both in the GUI and the Application Programming Interface (API). All features described in this article are available as open source software and are included in the latest release of the software [19].

In future work, we plan to add additional features to improve ARX's performance for high-dimensional data. While ARX already supports a wide range of data transformation models, we believe that the addition of further transformation methods would have the largest impact. One important example is sub-tree generalization, which provides a good balance between improved output data quality and interpretability of output datasets [58]. Moreover, we plan to add further methods from the area of statistical disclosure control, such as Post-Randomization (PRAM), that can be used to inject uncertainty into data with little impact on its usefulness [59].

Availability of supporting source code and requirements

Project name: ARX Anonymization Tool

Project home page: https://arx.deidentifier.org/

GitHub repository: https://github.com/arx-deidentifier/arx

Operating system(s): Platform independent

Programming language: Java 8

Other requirements: None

License: Apache License 2.0

RRID: SCR_021189

bio.tools ID: arx

Project name: Benchmark of ARX's Heuristic Algorithms

GitHub repository: https://github.com/arx-deidentifier/genetic-benchmark

Operating system(s): Platform independent

Programming language: Java 8, Python 3

Other requirements: None

License: Apache License 2.0

Data Availability

The datasets used to benchmark the algorithms are publicly available. The corresponding download URLs are referenced in Table 1 in Section 2.5.3. Additionally, the datasets are part of a GitHub repository containing our benchmarking code [60]. The repository also contains the generalization hierarchies used for anonymizing the data and the raw benchmark results as .csv files. A snapshot of the code and the supporting data is available in the *GigaScience* GigaDB repository [61].

Competing interests

The authors declare that there is no conflict of interest.

Funding

The authors received no specific funding for this work.

Author Contributions

R.B. initiated and conceptualized the work. F.P., K.D. and T.M. implemented the novel anonymization algorithms and integrated them into ARX. F.P. and T.M. reworked the user-interface of ARX. T.M. programmed the framework used to evaluate the novel algorithms and performed the benchmarks. T.M. and F.P. drafted the manuscript. R.B. and K.D. revised the manuscript and provided important suggestions for further improvements. All authors read and approved the final manuscript.

References

[1] S. Schneeweiss, "Learning from Big Health Care Data," *N. Engl. J. Med.*, vol. 370, no. 23, pp. 2161–2163, Jun. 2014, doi: 10.1056/NEJMp1401111.

[2] A. Ballantyne, "Where is the human in the data? A guide to ethical data use," *GigaScience*, vol. 7, no. 7, Jul. 2018, doi: 10.1093/gigascience/giy076.

[3] Office for Civil Rights, HHS, "Standards for privacy of individually identifiable health information. Final rule," *Fed. Regist.*, vol. 67, no. 157, pp. 53181–53273, Aug. 2002.

[4] "Regulation (EU) 2016/679 of the European Parliament and of the Council of 27 April 2016 on the protection of natural persons with regard to the processing of personal data and on the free movement of such data, and repealing Directive 95/46/EC (General Data Protection Regulation)".

[5] F. Ritchie, "Five Safes: designing data access for research," 2016, doi: 10.13140/RG.2.1.3661.1604.

[6] F. Prasser, J. Eicher, H. Spengler, R. Bild, and K. A. Kuhn, "Flexible data anonymization using ARX—Current status and challenges ahead," *Softw. Pract. Exp.*, vol. 50, no. 7, pp. 1277–1304, Jul. 2020, doi: 10.1002/spe.2812.

[7] B. Fung, K. Wang, A. W.-C. Fu, and P. Yu, *Introduction to privacy-preserving data publishing: Concepts and techniques*. 2010, p. 341. doi: 10.1201/9781420091502.

[8] L. Rocher, J. M. Hendrickx, and Y.-A. de Montjoye, "Estimating the success of re-identifications in incomplete datasets using generative models," *Nat. Commun.*, vol. 10, no. 1, p. 3069, Dec. 2019, doi: 10.1038/s41467-019-10933-3.

[9] L. Sweeney, "Simple Demographics Often Identify People Uniquely," *Carnegie Mellon University, Data Privacy*, 2000. [Online]. Available: http://dataprivacylab.org/projects/identifiability/

[10] A. Majeed and S. Lee, "Anonymization Techniques for Privacy Preserving Data Publishing: A Comprehensive Survey," *IEEE Access*, vol. 9, pp. 8512–8545, 2021, doi: 10.1109/ACCESS.2020.3045700.

[11] K. El Emam, E. Jonker, L. Arbuckle, and B. Malin, "A Systematic Review of Re-Identification Attacks on Health Data," *PLoS ONE*, vol. 6, no. 12, p. e28071, Dec. 2011, doi: 10.1371/journal.pone.0028071.

[12] J. Henriksen-Bulmer and S. Jeary, "Re-identification attacks—A systematic literature review," *Int. J. Inf. Manag.*, vol. 36, no. 6, pp. 1184–1192, Dec. 2016, doi: 10.1016/j.ijinfomgt.2016.08.002.

[13] C. Aggarwal, "On *k*-anonymity and the curse of dimensionality," in *Proc. 31st International Conference on Very Large Data Bases*, Trondheim, Norway, Aug. 2005, pp. 901–909.

[14] F. Prasser, F. Kohlmayer, and K. A. Kuhn, "Efficient and effective pruning strategies for health data de-identification," *BMC Med. Inform. Decis. Mak.*, vol. 16, no. 1, p. 49, Dec. 2016, doi: 10.1186/s12911-016-0287-2.

[15] K. Babu, N. Ranabothu, N. Kumar, M. Elliot, and S. Jena, "Achieving k-anonymity Using Improved Greedy Heuristics for Very Large Relational Databases," *Trans. Data Priv.*, vol. 6, pp. 1–17, Apr. 2013.

[16] J.-W. Byun, A. Kamra, E. Bertino, and N. Li, "Efficient k-Anonymization Using Clustering Techniques," in *Advances in Databases: Concepts, Systems and Applications*, vol. 4443, R. Kotagiri, P. R. Krishna, M. Mohania, and E. Nantajeewarawat, Eds. Berlin, Heidelberg: Springer Berlin Heidelberg, 2007, pp. 188–200. doi: 10.1007/978-3-540-71703-4_18.

[17] G. Loukides and J. Shao, "Clustering-Based K-Anonymisation Algorithms," in *Database and Expert Systems Applications*, vol. 4653, R. Wagner, N. Revell, and G. Pernul, Eds. Berlin, Heidelberg: Springer Berlin Heidelberg, 2007, pp. 761–771. doi: 10.1007/978-3-540-74469-6_74.

[18] H. Lee, S. Kim, J. W. Kim, and Y. D. Chung, “Utility-preserving anonymization for health data publishing,” *BMC Med. Inform. Decis. Mak.*, vol. 17, no. 1, p. 104, Dec. 2017, doi: 10.1186/s12911-017-0499-0.
[19] ARX Project (2021), *ARX Data Anonymization Tool*. (Version 3.9.0) [Computer software], https://github.com/arx-deidentifier/arx.
[20] “External guidance on the implementation of the European Medicines Agency policy on the publication of clinical data for medicinal products for human use (EMA/90915/2016 Version 1.4).” European Medicines Agency, Nov. 09, 2018.
[21] M. Elliot, E. Mackey, K. O’Hara, and C. Tudor, *The Anonymisation Decision-Making Framework*. UKAN, 2016.
[22] Lei Xu, Chunxiao Jiang, Yan Chen, Yong Ren, and K. J. R. Liu, “Privacy or Utility in Data Collection? A Contract Theoretic Approach,” *IEEE J. Sel. Top. Signal Process.*, vol. 9, no. 7, pp. 1256–1269, Oct. 2015, doi: 10.1109/JSTSP.2015.2425798.
[23] J. Kim, H. Ha, B.-G. Chun, S. Yoon, and S. K. Cha, “Collaborative analytics for data silos,” in *2016 IEEE 32nd International Conference on Data Engineering (ICDE)*, Helsinki, Finland, May 2016, pp. 743–754. doi: 10.1109/ICDE.2016.7498286.
[24] C. Costa, G. Chatzimilioudis, D. Zeinalipour-Yazti, and M. F. Mokbel, “Efficient Exploration of Telco Big Data with Compression and Decaying,” in *2017 IEEE 33rd International Conference on Data Engineering (ICDE)*, San Diego, CA, USA, Apr. 2017, pp. 1332–1343. doi: 10.1109/ICDE.2017.175.
[25] J. Kuzilek, M. Hlosta, and Z. Zdrahal, “Open University Learning Analytics dataset,” *Sci. Data*, vol. 4, no. 1, Art. no. 1, Nov. 2017, doi: 10.1038/sdata.2017.171.
[26] G. Ursin, S. Sen, J.-M. Mottu, and M. Nygård, “Protecting Privacy in Large Datasets—First We Assess the Risk; Then We Fuzzy the Data,” *Cancer Epidemiol. Biomarkers Prev.*, vol. 26, Jul. 2017, doi: 10.1158/1055-9965.EPI-17-0172.
[27] “Lean European Open Survey on SARS-CoV-2 Infected Patients - Studying SARS-CoV-2 collectively,” *Lean European Open Survey on SARS-CoV-2 Infected Patients*. https://leoss.net/ (accessed Jul. 09, 2020).
[28] L. Sweeney, “ACHIEVING k-ANONYMITY PRIVACY PROTECTION USING GENERALIZATION AND SUPPRESSION,” *Int. J. Uncertain. Fuzziness Knowl.-Based Syst.*, vol. 10, no. 05, pp. 571–588, Oct. 2002, doi: 10.1142/S021848850200165X.
[29] C. Dwork and A. Roth, “The Algorithmic Foundations of Differential Privacy,” *Found. Trends® Theor. Comput. Sci.*, vol. 9, no. 3–4, pp. 211–407, 2013, doi: 10.1561/0400000042.
[30] L. Sweeney, “Datafly: a system for providing anonymity in medical data,” in *Database Security XI*, T. Y. Lin and S. Qian, Eds. Boston, MA: Springer US, 1998, pp. 356–381. doi: 10.1007/978-0-387-35285-5_22.
[31] F. Prasser, R. Bild, J. Eicher, H. Spengler, and K. A. Kuhn, “Lightning: Utility-Driven Anonymization of High-Dimensional Data,” p. 25, 2016.
[32] J.-L. Lin and M.-C. Wei, “Genetic algorithm-based clustering approach for k-anonymization,” *Expert Syst. Appl.*, vol. 36, no. 6, pp. 9784–9792, Aug. 2009, doi: 10.1016/j.eswa.2009.02.009.
[33] K. El Emam *et al.*, “A Globally Optimal k-Anonymity Method for the De-Identification of Health Data,” *J. Am. Med. Inform. Assoc.*, vol. 16, no. 5, pp. 670–682, Sep. 2009, doi: 10.1197/jamia.M3144.
[34] F. Kohlmayer, F. Prasser, C. Eckert, A. Kemper, and K. A. Kuhn, “Highly efficient optimal k-anonymity for biomedical datasets,” in *2012 25th IEEE International Symposium on Computer-Based Medical Systems (CBMS)*, Rome, Italy, Jun. 2012, pp. 1–6. doi: 10.1109/CBMS.2012.6266366.
[35] F. Kohlmayer, F. Prasser, C. Eckert, A. Kemper, and K. A. Kuhn, “Flash: Efficient, Stable and Optimal K-Anonymity,” in *2012 International Conference on Privacy, Security, Risk and Trust and 2012 International Confernece on Social Computing*, Amsterdam, Netherlands, Sep. 2012, pp. 708–717. doi: 10.1109/SocialCom-PASSAT.2012.52.
[36] M. Mitchell, *An introduction to genetic algorithms*. Cambridge, Mass: MIT Press, 1996.

[37] S. Katoch, S. S. Chauhan, and V. Kumar, “A review on genetic algorithm: past, present, and future,” *Multimed. Tools Appl.*, vol. 80, no. 5, pp. 8091–8126, Feb. 2021, doi: 10.1007/s11042-020-10139-6.

[38] Z. Wan, Y. Vorobeychik, W. Xia, E. W. Clayton, M. Kantarcioglu, and B. Malin, “Expanding Access to Large-Scale Genomic Data While Promoting Privacy: A Game Theoretic Approach,” *Am. J. Hum. Genet.*, vol. 100, no. 2, pp. 316–322, Feb. 2017, doi: 10.1016/j.ajhg.2016.12.002.

[39] F. Prasser *et al.*, “An Open Source Tool for Game Theoretic Health Data De-Identification,” *AMIA Annu. Symp. Proc. AMIA Symp.*, vol. 2017, pp. 1430–1439, 2017.

[40] F. Prasser, F. Kohlmayer, and K. Kuhn, “The Importance of Context: Risk-based De-identification of Biomedical Data,” *Methods Inf. Med.*, vol. 55, no. 04, pp. 347–355, 2016, doi: 10.3414/ME16-01-0012.

[41] S. Sankararaman, G. Obozinski, M. I. Jordan, and E. Halperin, “Genomic privacy and limits of individual detection in a pool,” *Nat. Genet.*, vol. 41, no. 9, pp. 965–967, Sep. 2009, doi: 10.1038/ng.436.

[42] E. F. Webdev, “Eclipse Nebula - Supplemental Widgets for SWT,” *projects.eclipse.org*, Jan. 31, 2013. https://projects.eclipse.org/projects/technology.nebula (accessed Jul. 07, 2020).

[43] P. Samarati and L. Sweeney, “Generalizing data to provide anonymity when disclosing information (abstract),” in *Proceedings of the seventeenth ACM SIGACT-SIGMOD-SIGART symposium on Principles of database systems - PODS ’98*, Seattle, Washington, United States, 1998, p. 188. doi: 10.1145/275487.275508.

[44] *Sharing Clinical Trial Data: Maximizing Benefits, Minimizing Risk (Appendix B Concepts and Methods for De-identifying Clinical Trial Data)*. Washington, D.C.: National Academies Press, 2015, p. 18998. doi: 10.17226/18998.

[45] J. Pitman, “Random discrete distributions invariant under size-biased permutation,” *Adv. Appl. Probab.*, vol. 28, no. 2, pp. 525–539, Jun. 1996, doi: 10.2307/1428070.

[46] N. Hoshino, “Applying Pitman’s sampling formula to microdata disclosure risk assessment,” *J. Off. Stat.*, vol. 17, no. 4, p. 499, 2001.

[47] V. S. Iyengar, “Transforming data to satisfy privacy constraints,” in *Proceedings of the eighth ACM SIGKDD international conference on Knowledge discovery and data mining - KDD ’02*, Edmonton, Alberta, Canada, 2002, p. 279. doi: 10.1145/775047.775089.

[48] “UCI Machine Learning Repository: Adult Data Set.” http://archive.ics.uci.edu/ml/datasets/adult (accessed Jul. 07, 2020).

[49] “ATUS-X : ATUS Data Extract Builder.” https://www.atusdata.org/atus/index.shtml (accessed Jul. 07, 2020).

[50] “IPUMS NHIS.” https://nhis.ipums.org/nhis/ (accessed Jul. 07, 2020).

[51] U. C. Bureau, “American Community Survey (ACS),” *The United States Census Bureau*. https://www.census.gov/programs-surveys/acs (accessed Jul. 07, 2020).

[52] “UCI Machine Learning Repository: default of credit card clients Data Set.” https://archive.ics.uci.edu/ml/datasets/default+of+credit+card+clients (accessed Jul. 07, 2020).

[53] “Open psychology data: Raw data from online personality tests.” https://openpsychometrics.org/_rawdata/ (accessed Jul. 07, 2020).

[54] J. Casas-Roma, J. Herrera-Joancomartí, and V. Torra, “Comparing Random-Based and k-Anonymity-Based Algorithms for Graph Anonymization,” in *Modeling Decisions for Artificial Intelligence*, vol. 7647, V. Torra, Y. Narukawa, B. López, and M. Villaret, Eds. Berlin, Heidelberg: Springer Berlin Heidelberg, 2012, pp. 197–209. doi: 10.1007/978-3-642-34620-0_19.

[55] A. Solanas, A. Martinez-Balleste, J. M. Mateo-Sanz, and J. Domingo-Ferrer, “Multivariate Microaggregation Based Genetic Algorithms,” in *2006 3rd International IEEE Conference Intelligent Systems*, London, UK, Sep. 2006, pp. 65–70. doi: 10.1109/IS.2006.348395.

[56] Y. He and J. F. Naughton, “Anonymization of set-valued data via top-down, local generalization,” *Proc. VLDB Endow.*, vol. 2, no. 1, pp. 934–945, Aug. 2009, doi: 10.14778/1687627.1687733.

[57] B. C. M. Fung, Ke Wang, and P. S. Yu, “Top-Down Specialization for Information and Privacy Preservation,” in *21st International Conference on Data Engineering (ICDE’05)*, Tokyo, Japan, 2005, pp. 205–216. doi: 10.1109/ICDE.2005.143.
[58] B. C. M. Fung, K. Wang, R. Chen, and P. S. Yu, “Privacy-preserving data publishing: A survey of recent developments,” *ACM Comput. Surv.*, vol. 42, no. 4, pp. 1–53, Jun. 2010, doi: 10.1145/1749603.1749605.
[59] T. K. Nayak and S. A. Adeshiyan, “On Invariant Post-randomization for Statistical Disclosure Control: Invariant PRAM for Disclosure Control,” *Int. Stat. Rev.*, vol. 84, no. 1, pp. 26–42, Apr. 2016, doi: 10.1111/insr.12092.
[60] T. Meurers and F. Prasser (2021), *Benchmark of ARX’s Heuristic Algorithms*. [Computer software], GitHub, Retrieved 18.7.2021 from https://github.com/arx-deidentifier/genetic-benchmark.
[61] T. Meurers, R. Bild, K.-M. Do, and F. Prasser (2021), *Supporting data for “A scalable software solution for anonymizing high-dimensional biomedical data.”* GigaScience Database, http://dx.doi.org/10.5524/100929.

Figure1

Click here to access/download;Figure;Figure1.pdf

Age	Sex	ZIP	BMI	Diagnosis
55	M	92855	28.0	I47.1 Supraventricular tachycardia
91	M	94552	24.9	I50.1 Left ventricular failure
85	F	92855	26.7	I50.0 Congestive heart failure
68	F	94230	31.7	I47.1 Supraventricular tachycardia
77	F	92855	18.3	I47.0 Re-entry ventricular arrhythmia
82	F	92855	24.0	I50.0 Congestive heart failure
78	F	92301	28.1	I50.0 Left ventricular failure
75	M	94550	31.0	I47.0 Re-entry ventricular arrhythmia
82	M	94501	34.9	I47.0 Re-entry ventricular arrhythmia
69	M	93321	32.3	I50.1 Left ventricular failure

1 2 3 4 5 6

Age	Sex	ZIP	BMI	Diagnosis
81.7	F	92***	19-29	I50 Heart failure
81.7	F	92***	19-29	I50 Heart failure
81.7	F	92***	19-29	I50 Heart failure
75.0	*	94***	≥ 30	I47 Paroxysmal tachycardia
75.0	*	94***	≥ 30	I47 Paroxysmal tachycardia
75.0	*	94***	≥ 30	I47 Paroxysmal tachycardia

1 Sampling

2 Aggregation

3 Supression

4 Masking

5 Categorization

6 Generalization

Figure2
Click here to access/download;Figure;Figure2.pdf

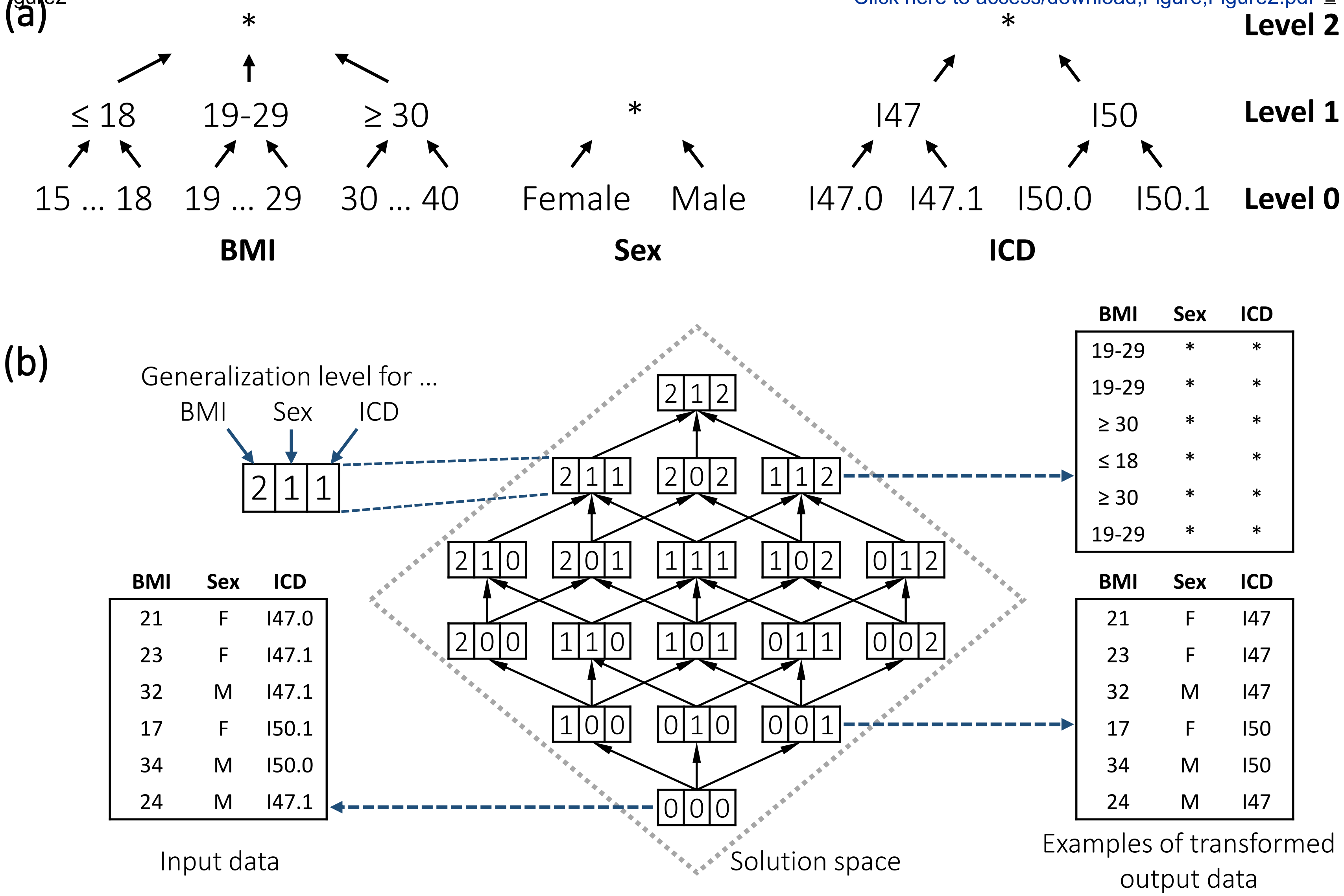

Figure3

Click here to access/download;Figure;Figure3.PNG

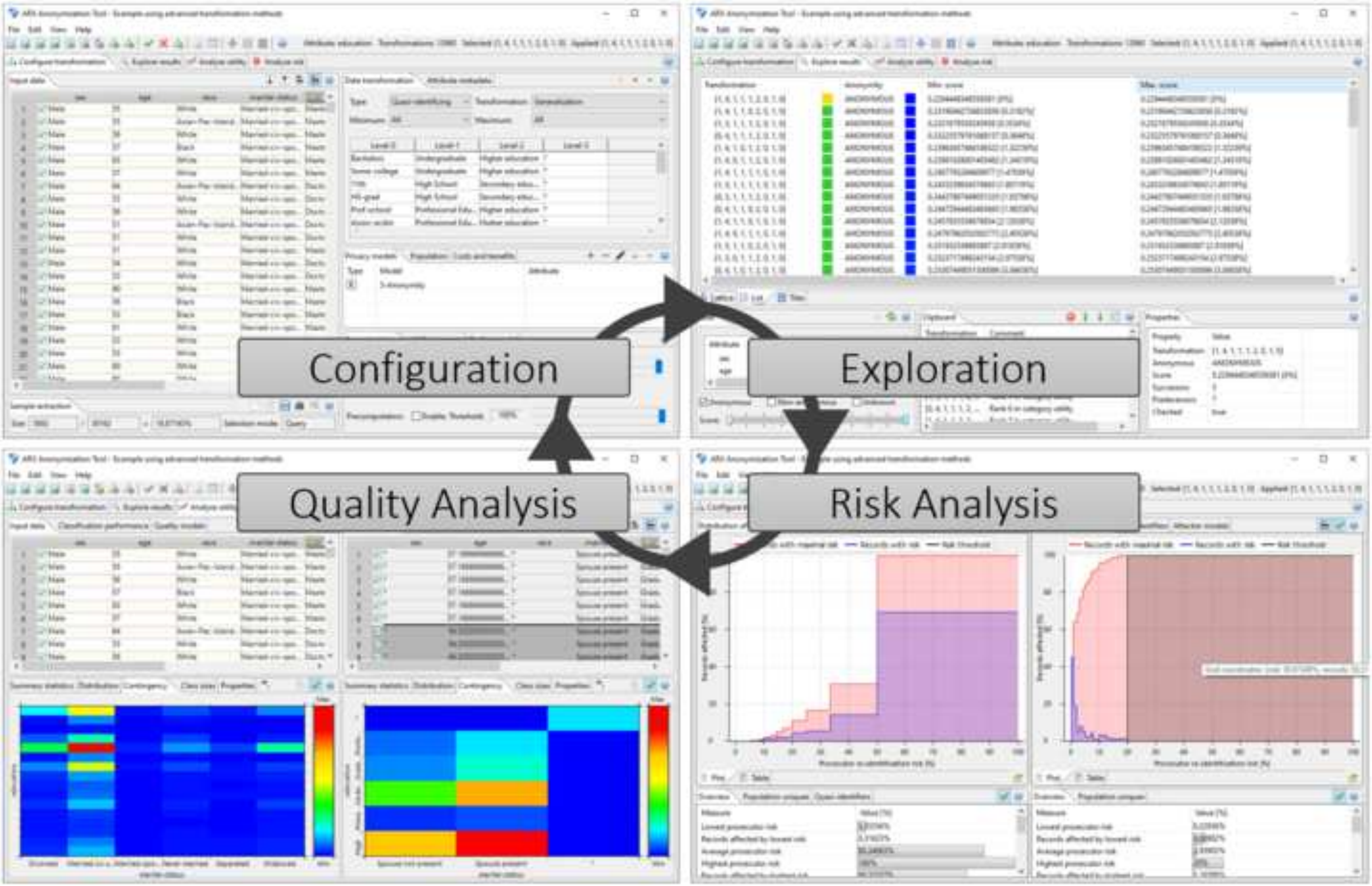

Figure4

Click here to access/download;Figure;Figure4.pdf

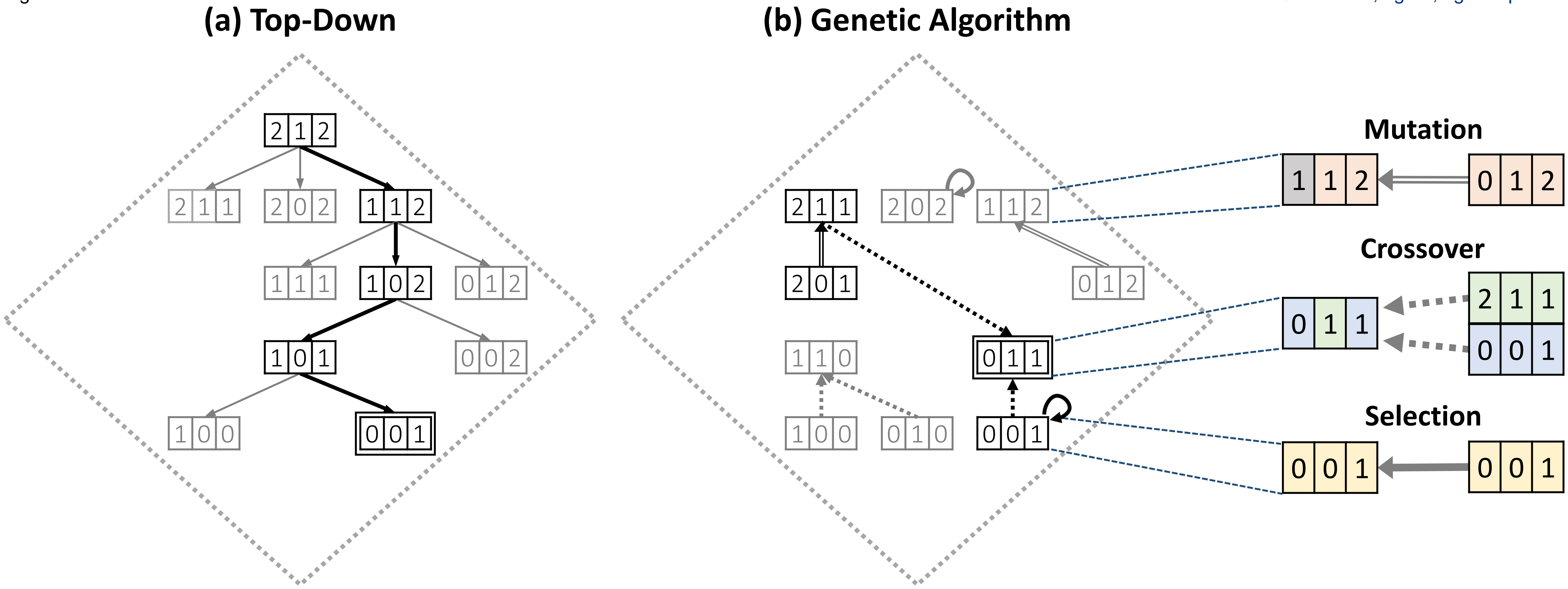

Figure5

Click here to access/download;Figure;Fig

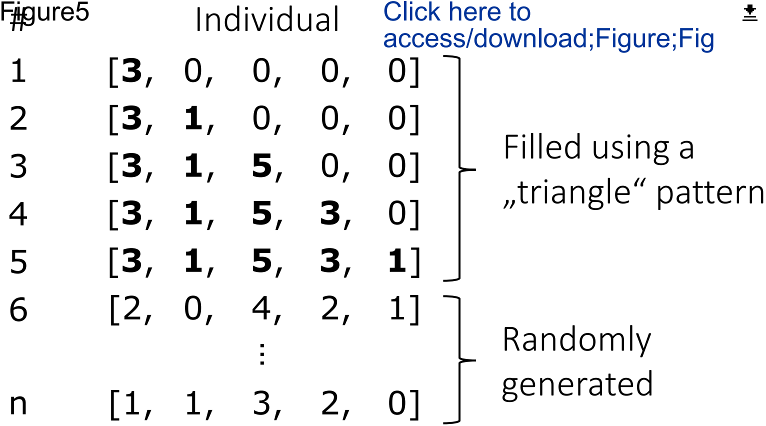

Figure6

Click here to access/download;Figure;Figure6.pdf

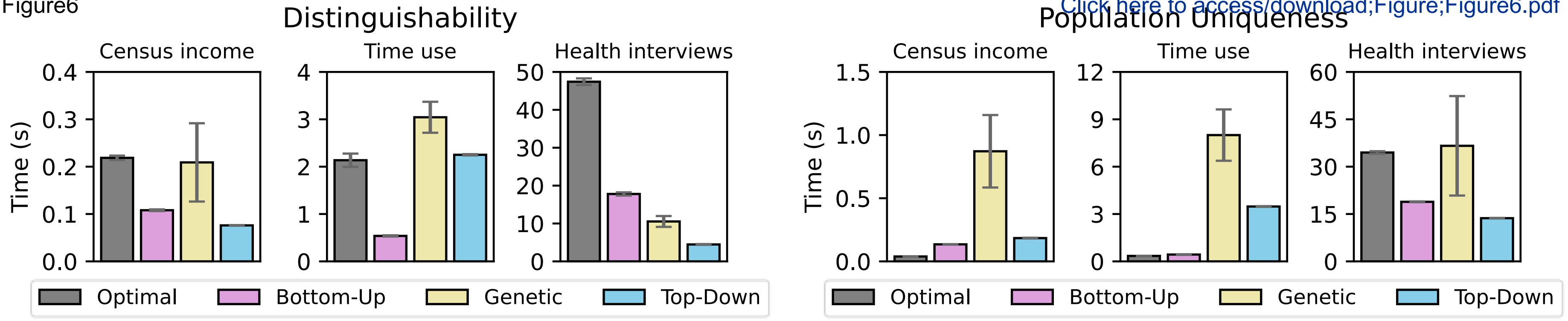

Figure7

Click here to access/download;Figure;Figure7.pdf

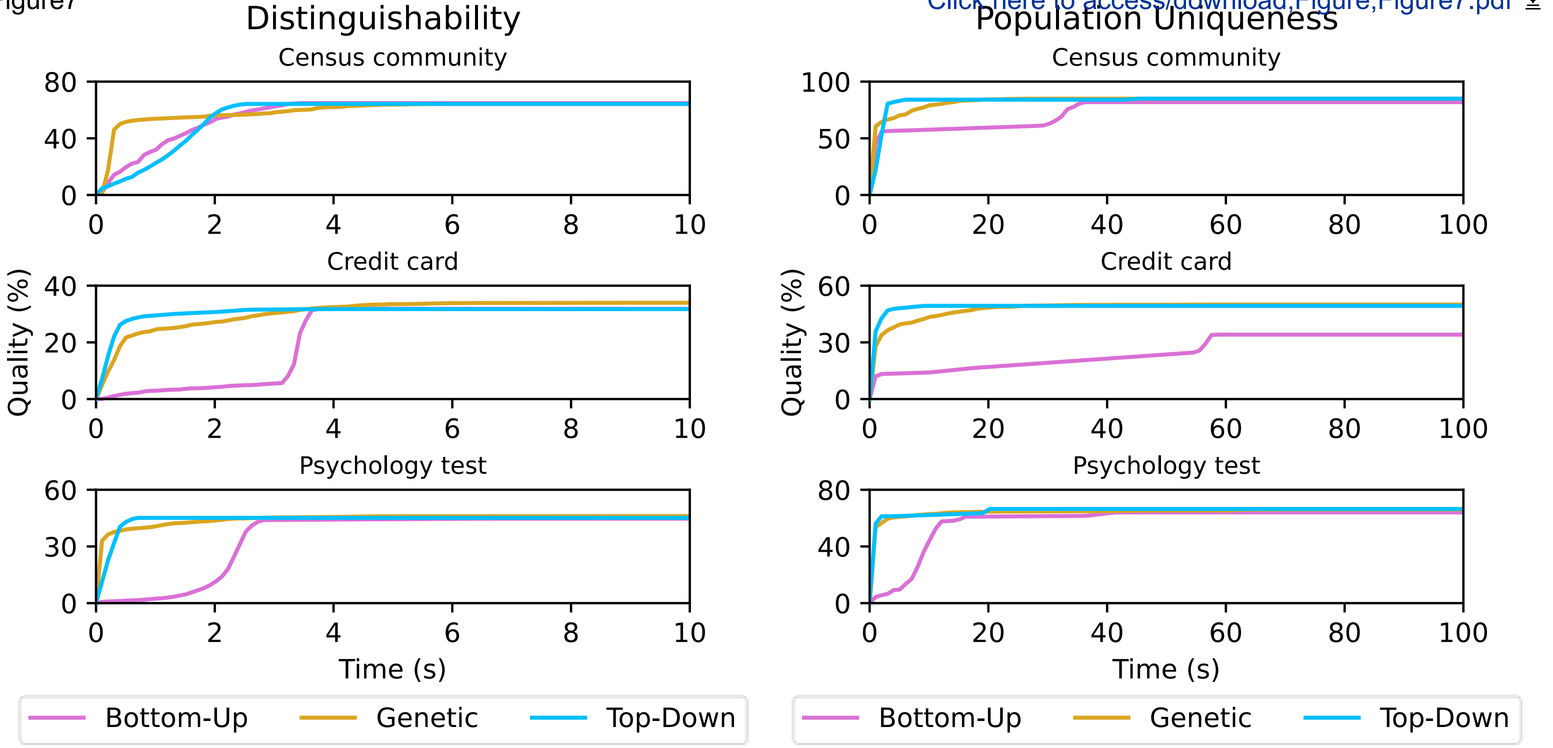

Figure8

Click here to access/download;Figure;Figure8.pdf

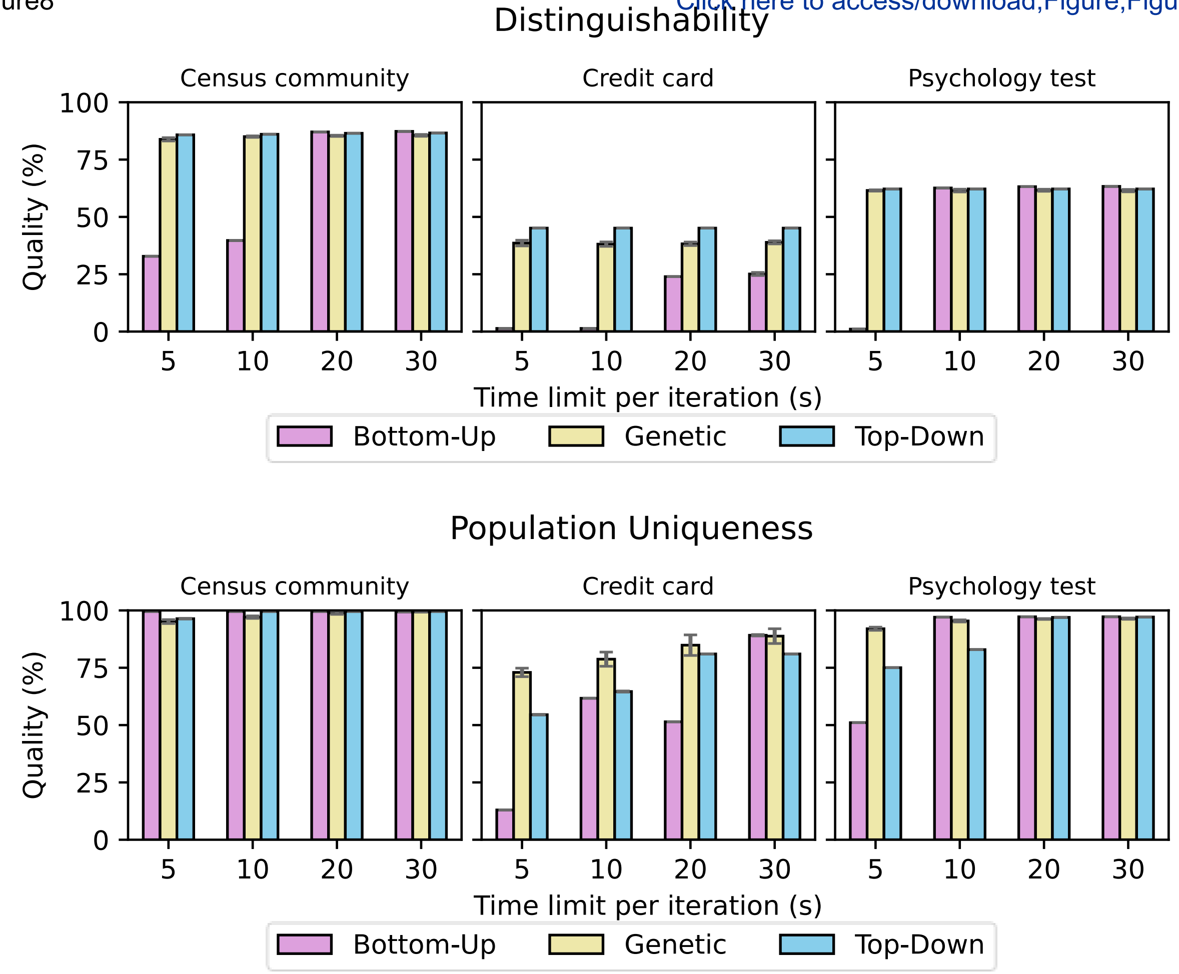

Figure9

Click here to access/download;Figure;Figure9.PNG

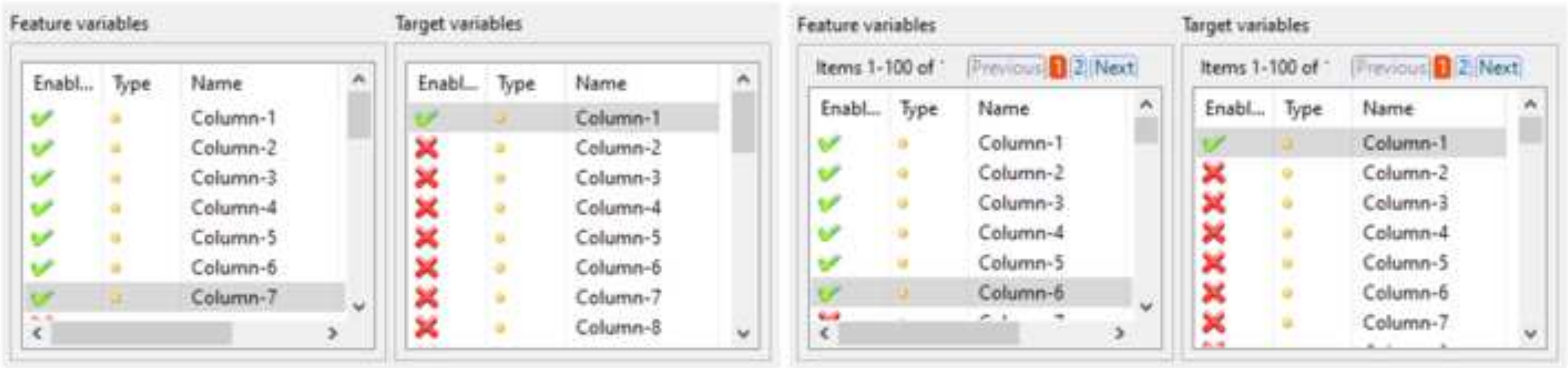

Click here to access/download
Supplementary Material
Supplementary File.pdf
