# Supplementary material for: A scalable software solution for anonymizing high-dimensional biomedical data
Source: Gigascience. 2021 Oct 4;10(10):giab068. doi: 10.1093/gigascience/giab068 (PMC8489190; doi:10.1093/gigascience/giab068)
Supplement: giab068_Supplemental_File [file giab068_supplemental_file.pdf]

Parameter Optimization

Variants of the genetic algorithm

Table S1: Results of the benchmark performed to compare variants of the genetic algorithm

Variant	Low-dimensional data (avg. execution time (s))	High-dimensional data (avg. utility (%))
Dual-Population + Triangle Pattern*†	16.7	75.3
Dual-Population	14.5	71.1
Single-Population	17.5	72.8

* Variant used in the final experiments
† Variant suggested by Wan et al.

Table S1 contains the results of the benchmark performed to compare different implementations of the genetic algorithm. We investigated three variants: approach 1 used a dual-population algorithm with "triangle pattern" (as proposed by Wan et al. [35]), approach 2 used a dual-population algorithm without "triangle pattern" and approach 3 used a single-population algorithm without "triangle pattern". For each variant we ran our set of experiments with low-dimensional data using global-generalization and our set of experiments with high-dimensional data using local-generalization (see Section 2.5 for a detailed description). We ran the experiments five times and reported the average time required to find an optimal solution, or, respectively, the average utility obtained.

Based on the experiments we opted for approach 1 (dual-population with "triangle pattern"). This variant performs well when processing low-dimensional data and significantly outperforms the other variants when processing high-dimensional datasets.

Parametrization of the genetic algorithm

Table S2: Results of the benchmark performed to optimize the parametrization of the genetic algorithm

Parameter	Value	Low-dimensional data (avg. execution time (s))	High-dimensional data (avg. utility (%))
eliteFraction	0.2*†	16.7	75.3
	0.4	19.0	75.5
crossoverFraction	0.2	18.6	75.4
	0.4*†	16.7	75.3
	0.6	17.7	75.3
productionFraction	0.2*	11.0	76.2
	0.4	10.9	76.1
	0.6	16.2	75.7
	0.8†	16.7	75.3
mutationProbability	0.025	16.3	75.3
	0.05*†	16.7	75.3
	0.1	16.6	75.4
immigrationFraction	0.2*†	16.7	75.3
	0.4	19.7	75.5
	0.6	20.6	75.3
	0.8	18.6	75.5
immigrationInterval	5	19.9	75.6
	10*†	16.7	75.3
	20	19.3	75.4
subpopulationSize	50*	14.0	75.9
	100‡	16.7	75.3
	200	27.3	74.3

* Configuration used in the final experiments
† Configuration suggested by Wan et al. and used as default
‡ Configuration used as default

Table S2 contains the results of the benchmark performed to optimize the parametrization of the genetic algorithm. Evaluating the parametrization was done as follows: (1) we started with the recommendations made by Wan et al. [35], and, (2) performed a systematic analysis of the influence of parameters by individually altering them and running the experiments. For each parametrization we ran our set of experiments with low-dimensional data using global-generalization and our set of experiments with high-dimensional data using local-generalization (see Section 2.5 for a detailed description). We ran the experiments five times and reported the average time required to find an optimal solution, or, respectively, the average utility obtained.
For parameters representing a fraction we evaluated the values $\{0.2, 0.4, 0.6, 0.8\}$. For the remaining parameters we evaluated values that correspond either to the half or to the double of the value suggested by Wan et al. The only exception was the *subpopulationSize*. The approach of Wan et al. of determining the size of the population is specifically tailored towards genetic data and is derived from the number of single-nucleotide polymorphisms (SNPs) available for sharing. Therefore, we decided to choose a default of 100 as population size, as this value is often used in literature [33] and is also suggested by Solans et al. [52].
Based on the preliminary experiments we set the $productionFraction$ to 0.2 (instead to 0.8 as suggested by Wan et al.) and opted for a $subpopulationSize$ of 50 individuals. Using these parameters led to good performance when processing low- as well as high-dimensional data in our experiments.

Note: The genetic algorithm populates each new generation with individuals using three different mechanisms: selection, crossover and mutation. The fraction of individuals resulting from selection and crossover can explicitly be adjusted using the $eliteFraction$ and the $corssoverFraction$;

The fraction of mutated individuals is implicitly derived from the remainder of these parameters ($mutationFraction = 1 - (eliteFraction + crossoverFraction)$). For this reason, we made sure that the $eliteFraction$ and the $crossoverFraction$ did not add up to a value ≥ 1 as this would mean that no mutation would be performed.
