# Supplementary material for: A scalable software solution for anonymizing high-dimensional biomedical data
Source: Gigascience. 2021 Oct 4;10(10):giab068. doi: 10.1093/gigascience/giab068 (PMC8489190; doi:10.1093/gigascience/giab068)
Supplement: giab068_Response_to_Reviewer_Comments_Original_Submission [file giab068_response_to_reviewer_comments_original_submission.pdf]

Author's Response To Reviewer Comments

Close

Dear Dr. Edmunds,

we are pleased to submit a revised version of our manuscript GIGA-D-20-00292R1 entitled “A scalable software solution for anonymizing high-dimensional biomedical data" by Thierry Meurers, Raffael Bild, Kieu-Mi Do and Fabian Prasser for consideration for publication in GigaScience.

We thank the reviewers and the editor for the helpful and constructive feedback. The most important changes to our manuscript are:

1) We added an additional figure (Figure 4) to Section 2.3 (“Integrating Anonymization Algorithms for High-Dimensional Data”), which illustrates how the novel algorithms search the solution space.
2) We have added a new subsection (Section 4.2 “Limitations”) to the discussion section in which we describe limitations of our study.
3) We have more clearly emphasized the contributions of our work in sections “Introduction”, “Principal results” and “Comparison with prior work”.
4) We have provided more details on why we chose to implement a genetic algorithm, how we decided on its exact design and how we determined the parameterization used in the experiments.
5) We have revised various parts of the manuscript to address minor comments by the editor and the reviewers and performed some editorial changes to improve the readability of the paper.

A detailed overview of our changes regarding the reviewers’ comments is listed in the point-by-point-response below.

With kind regards,
Thierry Meurers

Editor (Scott Edmunds)

Comment 1: Please register any new software application in the bio.tools and SciCrunch.org databases to receive RRID (Research Resource Identification Initiative ID) and biotoolsID identifiers, and include these in your manuscript. This will facilitate tracking, reproducibility and re-use of your tool. Please also follow the software citation guidelines for software discussed here https://f1000research.com/articles/9-1257/v2

Response: We thank the editor for pointing this out. We registered ARX to bio.tools (https://bio.tools/arx) and SciCrunch.org (SCR_021189) and included the IDs in the “Availability of supporting source code and requirements” Section of the revised manuscript. Furthermore, we changed the citation of ARX to comply to the software citation guidelines mentioned.

Reviewer #1

Comment 1: The manuscript is clear, and well-written overall. The manuscript has sufficient originality, and undertaken problem is of practical nature. Although the results presented in the manuscript seem promising and overall approach is contributing to the body of the literature, I encourage the authors to please consider the attached file suggestions/comments to improvise the presented work more prior to its publication.

Response: We thank the reviewer for this positive feedback! We have addressed the individual comments contained in the attached file as described below.

Comment 2: In figure 1, I would recommend adding a one comprehensive in terms of data input overview, intermediate steps with two proposed methods illustration or formalization, and final output after passing through all steps.

Response: We thank the reviewer for this very good suggestion. Figure 1 is part of Section 2.1 ("Fundamentals of Data Anonymization") and it is meant to only provide a general overview of how data can be altered in the context of anonymization. For this reason, we added an additional figure (Figure 4) to Section 2.3 ("Integrating Anonymization Algorithms for High-Dimensional Data"), which illustrates how the novel algorithms search the solution space.

Comment 3: In my opinion, section I should include some more recent and pertinent studies. Authors can possibly include the recent and closely related studies,
- Majeed, A., & Lee, S. (2020). Anonymization Techniques for Privacy Preserving Data Publishing: A Comprehensive Survey. IEEE Access.
- Majeed, A., & Lee, S. (2020). Attribute susceptibility and entropy based data anonymization to improve users community privacy and utility in publishing data. Applied Intelligence, 1-20.
- Majeed, A., Ullah, F., & Lee, S. (2017). Vulnerability-and diversity-aware anonymization of personally identifiable information for improving user privacy and utility of publishing data. Sensors, 17(5), 1059

Response: We thank the reviewer for providing this list of recent studies in the field. We included the first study listed (survey article by Majeed & Lee) to the manuscript's introduction section (Section 1), where it fits well as it is a recent survey on the topic.

Comment 4: The limitations of the presented study are not provided by the authors. It would be better to highlight them in the revised work.

Response: We agree with the reviewer that more information on the limitations of our work were needed. We have added a corresponding section to the discussion section (Section 4.2 "Limitations") in which we address 1) the dependence of our benchmark results on the datasets and privacy models used, as well as 2) the difficulty of properly parameterizing genetic algorithms in general.

Comment 5: Some more and pertinent keywords can be added related to subject matter presented in this paper.

Response: We thank the reviewer for this suggestion. We added three additional keywords (privacy preserving data publishing, biomedical data, data protection) to further describe the paper's subject.

Comment 6: All symbols can be written in math mode for better readability of the manuscript.

Response: We thank you for this suggestion. We replaced all symbols in the manuscript using the math mode.

Comment 7: Some examples and scenarios that emphasize the technical contribution of this paper can be explained in the revised work.

Response: We thank the reviewer for pointing out that this required further clarification. We rewrote parts of Section 4.1 ("Principal results") to provide a better understanding of scenarios where using the new algorithms can be beneficial: "Evaluating the newly implemented algorithms showed that they are particularly useful in scenarios where high-dimensional data needs to be anonymized." Furthermore, we added a synthesis of our results to underline the most important results (see the next comment below). To highlight the novelty of our work in the context of genetic anonymization algorithms, we rewrote Section 4.3 ("Comparison with Prior Work"): "It has been demonstrated multiple times that genetic algorithms can be used for anonymizing data. However, previously described solutions were mostly tailored towards specific types of data or privacy and transformation models. […] Our work is different from these approaches, because it integrates a genetic algorithm into ARX in such a way that it can be

used to anonymize datasets using a variety of privacy models, quality models and data transformation schemes.”.
Finally, we have also added a concise numbered list of our contributions to the introduction section (see also our reply to Comment 11 below).

Comment 8: Some detailed synthetises of the results obtained from experiments can be provided for the better readership of this article before conclusion section.

Response: We thank the reviewer for this suggestion. We rewrote parts of Section 4.1.(“Principal Results”) to provide a more thorough synthesis the results obtained: “Evaluating the newly implemented algorithms showed that they are particularly useful in scenarios where high-dimensional data needs to be anonymized. Using global generalization, they clearly outperformed the previously implemented bottom-up search (i.e. better performance in 5 of the 6 experiments). A similar result was observed when using local generalization. Averaged over all experiments, the new algorithms achieved a utility of 76.5 % (genetic algorithm) and 75.1 % (top-down algorithm), which is significantly higher than that provided by the bottom-up approach (60.2%). Especially when anonymizing the dataset with the largest solution space (credit card), the new algorithms often performed significantly better, both in terms of scalability and utility. Additionally, the results obtained when processing low-dimensional data showed that heuristic algorithms can be helpful to improve computational efficiency even in scenarios where optimal algorithms could be used. The top-down approach required the least amount of time on average to find an optimal solution (4.0 s), followed by the bottom-up approach (6.3 s), the genetic algorithm (9.9 s) and the optimal search strategy (14.1 s).”

Comment 9: All figures must be placed on the appropriate position in the revised work.

Response: Thanks for pointing this out. We carefully revised the positioning of our figures and moved one figure (Fig. 5) closer to the corresponding text in the manuscript. Furthermore, we have noticed that one of the figures was wrongly numbered and fixed this as well. Following the author guidelines of GigaScience, all figures are submitted in separate files and their position within the main manuscript is indicated using placeholders and captions.

Comment 10: This manuscript categorization can be written concisely in the introduction section of the revised work.

Response: We thank the reviewer for this suggestion. We added the type of our manuscript (Technical Note) to the introduction (Section 1).

Comment 11: Contribution can be presented with bullets concisely in the revised work.

Response: Thank you for this suggestion. We rewrote the end of the introduction (Section 1) to provide a concise overview of our contributions which are: “(1) extending ARX’s user interface with additional views that simplify the management of high-dimensional data, (2) implementing two novel heuristic anonymization algorithms and (3) evaluating the novel algorithms regarding their performance for anonymizing low-dimensional and high-dimensional datasets.”

Reviewer #2

Comment 1: This is a very interesting and valuable result. However, there are several concerns.

Response: We thank the reviewer for this kind and positive feedback! We have addressed the concerns raised as described below.

Comment 2: There is not enough convincing explanation for the reason for applying genetic algorithm. There are several methods for optimization including simulated annealing. Moreover, as the author mentioned, there was already an attempt to apply genetic algorithm for data anonymization (Wan et

al.). To promote the novelty of the presenting study, it needs to provide more robust explanation for selecting genetic algorithm for this project.

Response: We thank the reviewer for pointing out that this needed to be clarified. We integrated the genetic algorithm into ARX as it is a well-known population-based metaheuristic. In contrast to single-solution based approaches (like, for example, greedy heuristics or simulated annealing) multiple candidate solutions are maintained which results in a high degree of diversification and lowers the risk of getting stuck in local optima. We added these details to Section 2.3 ("Integrating Anonymization Algorithms for High-Dimensional Data") of the revised manuscript.
Other papers using genetic algorithms for data anonymization are tailored towards a specific type of data (e.g. the approach implemented by Wan et al. is exclusively used for anonymizing genetic data, which is a process that is very different from the process focused on in our work) or only allow for specific anonymization procedures (e.g. by partitioning the data to fulfill k-anonymity). Integrating a genetic algorithm into the generic data anonymization core provided by ARX enables using a genetic anonymization algorithm for a wide range of tabular datasets using a wide range of privacy models and data transformation methods. We have added these additional aspects highlighting the novelty of our work to Section 2.3 ("Integrating Anonymization Algorithms for High-Dimensional Data") when introducing the algorithm and also rewrote parts of Section 4.3 ("Comparison to prior work") to make this clearer.

Comment 3. Genetic algorithm is little bit complicate to handle. Author showed the detailed parameter setting for the present study. It is necessary to provide a reference as well as scientific reasons for setting parameters. In addition, why do the authors use 'triangle pattern' in genetic algorithm for this research?

Response: We agree with the reviewer that these points require a careful consideration. Both, the parametrization as well as the 'triangle pattern' were inspired by the work of Wan et al.. For our first submission, we run preliminary benchmarks in which we individually varied a subset of the parameters, which resulted in setting the productionFraction to 0.2 (instead to 0.8 as suggested by Wan et al.). For the new revision of the manuscript, we conducted a systematic evaluation of all parameters and also checked whether the 'triangle pattern' is advantageous for our use case.
Evaluating the parametrization was done as follows: (1) we started with the recommendations made by Wan et al., and, (2) performed a systematic analysis of the influence of parameters by individually altering them and running the evaluation benchmarks. The detailed procedure and its results are described in a supplementary file (see Supplementary Table S2) which is also referenced in the revised version of Section 2.5.3 ("Parametrization"). The results confirmed that setting the productionFraction to 0.2 improves the algorithm's performance in our experiments. Additionally, the preliminary experiments also showed that decreasing the subpopulationSize from 100 to 50 leads to further improvements in our setting. Although the difference caused by the reduction of the subpopulationSize was only small, we repeated our experiments with this configuration and updated the figures in the paper.
Moreover, we performed another set of experiments to compare different variants of the genetic algorithm to motivate the use of the "triangle pattern". We investigated three variants: approach 1 used a dual-population algorithm with "triangle pattern" (as proposed by Wan et al.), approach 2 used a dual-population algorithm without "triangle pattern" and approach 3 used a single-population algorithm without "triangle pattern".
Running our experiments showed that approach 1 ("triangle pattern" with dual-population) offers the best average performance. This can be explained by the triangle-pattern covering a larger part of the overall solution space in the beginning. We added this explanation to Section 2.3 ("Integrating Anonymization Algorithms for High-Dimensional Data") when explaining the initialization procedure and included the results of the preliminary benchmark in the supplementary file (see Supplementary Table S1).

Comment 4: Authors introduced several approaches (#1. top-down #2. bottom-up #3. genetic algorithm). Genetic algorithm showed better performance for the complex and high dimensional datasets but lower performance for the low dimensional datasets. If the authors propose some guidance to select the appropriate algorithm for data anonymization, it would be very helpful for general users of ARX solution.

Response: We thank the reviewer for this suggestion. Generally recommending a specific algorithm is difficult due to the performance being highly dependent on the dataset anonymized and the

configuration utilized. Nevertheless, we revised Section 4.1 ("Principal results") to contain a concise summary of our evaluation results and highlighted ARX's capability of automatically deciding whether it is feasible to calculate an optimal solution or whether a heuristic search is required. Furthermore, we mentioned that ARX provides means to easily try out different algorithms and compare their results to facilitate the selection of a method well suited in a specific context.

Close
