# Supplementary material for: A scalable software solution for anonymizing high-dimensional biomedical data
Source: Gigascience. 2021 Oct 4;10(10):giab068. doi: 10.1093/gigascience/giab068 (PMC8489190; doi:10.1093/gigascience/giab068)
Supplement: giab068_Response_to_Reviewer_Comments_Revision_1 [file giab068_response_to_reviewer_comments_revision_1.pdf]

Author's Response To Reviewer Comments

Close

--- Reviewer 1 ---:
Comment 1: The manuscript has significantly improved.

Response: We thank the reviewer for this kind feedback! The remaining comments are addressed below.

Comment 2: Meanwhile, it would be better to restructure the contents. For example, all figures at the end are not desirable, and are hard to understand.

Response: We thanks the reviewer for pointing this out. We carefully revised the structure of the document and decided to move Figure 4, to include a more thorough description of the figure and to move Section 2.5.3 (“Datasets”) as well as the table within this section. We would like to point out that all figures were uploaded separately to comply with the journal’s submission guideline. This is the reason for them currently appearing at the end of the document. However, their final positions in the manuscript are indicated by placeholders and captions, so they can be moved to their correct position during typesetting.

Comment 3: Also, it would be better to discuss the details of high and low dimensional data.

Response: We thank the reviewer and agree that this needed further clarification. We revised Section 1 (“Introduction”) to describe more clearly what we mean by low- and high-dimensional and to emphasize the challenges related to the anonymization of high-dimensional data. Furthermore, we added more details about the dimensionality of the datasets used to Section 2.5.1 (“Experiments”).

Comment 4: Please modify the comprehensive figure to highlight the complete workflow of the approach with data.

Response: We thank you for this suggestion. To improve the illustration of our approach, we decided to revise two Figures (2 and 4) and to add additional explanations. Figure 2 now illustrates how an exemplary dataset can be transformed by applying different generalization schemes from the solution space. We also revised Figure 4 to be directly based on the example presented in Figure 2 to convey a more complete picture. We also revised and extended the explanation of the genetic algorithm in Section 2.3 (“Integrating Anonymization Algorithms for High-Dimensional Data”), which now relates to the example provided in Figure 4.

Comment 5: A final version should depict the good structure of the paper that can be perceived easily by the readers.

Response: We would like to thank the reviewer once again for the important comments regarding the structure. We have reviewed the structure thoroughly, made the changes described above and are optimistic that the reading flow has improved significantly (especially when all the illustrations are moved to their final position during typesetting).

--- Reviewer 2 ---:
Comment 1: The authors have responded appropriately to the issues raised, and the manuscript has been modified accordingly. There are no further queries.

Response: We thank the reviewer for this positive feedback and for the valuable comments made on the previous version of the manuscript!

--- Editor ---:
Comment 1: [The reviewers] have requested a final check of the formatting,

Response: As described in our response to the comments by reviewer 1, we have thoroughly checked

the structure and formatting of the paper to improve its reading flow. We believe that the manuscript has improved significantly in this regard.

Comment 2: and we would also recommend you follow the software citation guidelines for all software mentioned in the paper: https://academic.oup.com/gigascience/pages/technical_note

Response: Thank you for pointing this out. We checked all references to software and revised the citation of ARX and included a new to our benchmark code.

Comment 3: I've cc'd our curators here who will help you archive a snapshot of the code and any other materials in our GigaDB repository, giving it a DOI that you will need to cite in an Data Availability section in the paper.

Response: Thanks to the help of the curators, a snapshot of our supporting code and data was uploaded to a GigaDB repository. We added a reference for the DOI and included it in the Data Availability section.

Close
