# Supplementary material for: A scalable software solution for anonymizing high-dimensional biomedical data
Source: Gigascience. 2021 Oct 4;10(10):giab068. doi: 10.1093/gigascience/giab068 (PMC8489190; doi:10.1093/gigascience/giab068)
Supplement: giab068_Reviewer_2_Report_Original_Submission — Hyung Joon JOO -- 4/21/2021 Reviewed [file giab068_reviewer_2_report_original_submission.pdf]

Reviewer Report

Title: A scalable software solution for anonymizing high-dimensional biomedical data

Version: Original Submission **Date:** 4/21/2021

Reviewer name: Hyung Joon JOO

Reviewer Comments to Author:

This is a very interesting and valuable result. However, there are several concerns.
1. There is not enough convincing explanation for the reason for applying genetic algorithm. There are several methods for optimization including simulated annealing.
Moreover, as the author mentioned, there was already an attempt to apply genetic algorithm for data anonymization (Wan et al.). To promote the novelty of the presenting study, it needs to provide more robust explanation for selecting genetic algorithm for this project.
2. Genetic algorithm is little bit complicate to handle. Author showed the detailed parameter setting for the present study. It is necessary to provide a reference as well as scientific reasons for setting parameters. In addition, why do the authors use 'triangle pattern' in genetic algorithm for this research?
3. Authors introduced several approaches (#1. top-down #2. bottom-up #3. genetic algorithm). Genetic algorithm showed better performance for the complex and high dimensional datasets but lower performance for the low dimensional datasets. If the authors propose some guidance to select the appropriate algorithm for data anonymization, it would be very helpful for general users of ARX solution.

Level of Interest

Please indicate how interesting you found the manuscript: Choose an item.

Quality of Written English

Please indicate the quality of language in the manuscript: Choose an item.

Declaration of Competing Interests

Please complete a declaration of competing interests, considering the following questions:

- Have you in the past five years received reimbursements, fees, funding, or salary from an organisation that may in any way gain or lose financially from the publication of this manuscript, either now or in the future?
- Do you hold any stocks or shares in an organisation that may in any way gain or lose financially from the publication of this manuscript, either now or in the future?
- Do you hold or are you currently applying for any patents relating to the content of the manuscript?

- Have you received reimbursements, fees, funding, or salary from an organization that holds or has applied for patents relating to the content of the manuscript?
- Do you have any other financial competing interests?
- Do you have any non-financial competing interests in relation to this paper?

If you can answer no to all of the above, write 'I declare that I have no competing interests' below. If your reply is yes to any, please give details below.

I declare that I have no competing interests'

I agree to the open peer review policy of the journal. I understand that my name will be included on my report to the authors and, if the manuscript is accepted for publication, my named report including any attachments I upload will be posted on the website along with the authors' responses. I agree for my report to be made available under an Open Access Creative Commons CC-BY license (http://creativecommons.org/licenses/by/4.0/). I understand that any comments which I do not wish to be included in my named report can be included as confidential comments to the editors, which will not be published.

Choose an item.

To further support our reviewers, we have joined with Publons, where you can gain additional credit to further highlight your hard work (see: https://publons.com/journal/530/gigascience). On publication of this paper, your review will be automatically added to Publons, you can then choose whether or not to claim your Publons credit. I understand this statement.

Yes Choose an item.
